# Characterization of the rumen lipidome and microbiome of steers fed a diet supplemented with flax and echium oil

**DOI:** 10.1111/1751-7915.12164

**Published:** 2014-09-16

**Authors:** Sharon Ann Huws, Eun Jun Kim, Simon J S Cameron, Susan E Girdwood, Lynfa Davies, John Tweed, Hannah Vallin, Nigel David Scollan

**Affiliations:** 1 Institute of Biological, Environmental and Rural Sciences (IBERS), Aberystwyth UniversityPenglais Campus, Aberystwyth, SY23 3DA, UK; 2 Hybu Cig Cymru – Meat Promotion WalesTy Rheidol Parc Merlin, Aberystwyth, UK

## Abstract

Developing novel strategies for improving the fatty acid composition of ruminant products relies upon increasing our understanding of rumen bacterial lipid metabolism. This study investigated whether flax or echium oil supplementation of steer diets could alter the rumen fatty acids and change the microbiome. Six Hereford × Friesian steers were offered grass silage/sugar beet pulp only (GS), or GS supplemented either with flax oil (GSF) or echium oil (GSE) at 3% kg^−1^ silage dry matter in a 3 × 3 replicated Latin square design with 21-day periods with rumen samples taken on day 21 for the analyses of the fatty acids and microbiome. Flax oil supplementation of steer diets increased the intake of polyunsaturated fatty acids, but a substantial degree of rumen biohydrogenation was seen. Likewise, echium oil supplementation of steer diets resulted in increased intake of 18:4*n*-3, but this was substantially biohydrogenated within the rumen. Microbiome pyrosequences showed that 50% of the bacterial genera were core to all diets (found at least once under each dietary intervention), with 19.10%, 5.460% and 12.02% being unique to the rumen microbiota of steers fed GS, GSF and GSE respectively. Higher 16S rDNA sequence abundance of the genera *Butyrivibrio*, *Howardella*, *Oribacterium*, *Pseudobutyrivibrio* and *Roseburia* was seen post flax feeding. Higher 16S rDNA abundance of the genus *Succinovibrio* and *Roseburia* was seen post echium feeding. The role of these bacteria in biohydrogenation now requires further study.

## Introduction

Ruminant animals exclusively supply all dairy products and *c.* 50% of meat consumed globally (Meat Promotion Wales, pers. comm.), and so are a vital component of the human diet. Nonetheless, due to a growing population and a nutrition transition towards increased intake of livestock products, demand for such products will increase dramatically over the coming decades (Foresight, 2011). Ruminants are able to convert plant biomass to chemical compounds, which are subsequently metabolized and absorbed by the animal, largely due to the functional capacity of their diverse rumen microbiota (Mackie, 2002; Edwards *et al*., 2008; Kingston-Smith *et al*., 2010). Indeed, the fermentative capacity of the rumen microbiota defines the amount, quality, and composition of meat and milk (Edwards *et al*., 2008; Kingston-Smith *et al*., 2010).

Ruminant products are considered detrimental for human health due to their high levels of saturated fatty acid (SFA). Forage lipids are rich in polyunsaturated fatty acids (PUFA), particularly 18:3*n-3*, which are beneficial to human health, yet these are only partially transferred into meat and milk (Scollan *et al*., 2006; Jenkins *et al*., 2008; Lourenço *et al*., 2010). This is due to the action of the rumen microbiota, which biohydrogenate dietary PUFA to SFA, producing transitionary conjugated diene and triene, as well as monoene intermediates (Huws *et al*., 2010; 2011; Lourenço *et al*., 2010). In recent years, much emphasis has been given to developing novel strategies of controlling biohydrogenation to enhance the health benefits of ruminant products for the consumer. Developing these strategies requires a greater level of understanding of the role of the rumen microbiota in biohydrogenation. Denaturing gradient gel electrophoresis has demonstrated that many as yet uncultured rumen bacteria belonging to the genera *Prevotella*, Lachnospiraceae incertae sedis, and unclassified Bacteroidales, Clostridiales and Ruminococcaceae, may have biohydrogenating capacity (Boeckaert *et al*., 2008; Kim *et al*., 2008; Belenguer *et al*., 2010; Huws *et al*., 2011). With the advent of next-generation sequencing, we are now able to probe the possible linkages between the lipidome and microbiome, which may be due to biohydrogenation capacity, for further testing.

Attempts to improve the fatty acid quality of meat and milk to date have been untargeted, due to our limited understanding of bacterial biohydrogenation, and based upon many strategies, including plant-based strategies such as the use of tannins, phenols and saponins (Edwards *et al*., 2008), but mainly based on using oil supplementation. The underlying hypothesis in terms of oil-based strategies is that by increasing the intake of beneficial fatty acids, more fatty acids reach the duodenum and subsequently are incorporated in meat and milk. There is a wealth of knowledge showing that flax (*Linum usitatissimum*) and fish oil supplementation of the ruminant diet increases the absorption of fatty acids, which have beneficial health properties, e.g. PUFA, conjugated linoleic acid (CLA) and 18:1 *trans*-11 flow (Lee *et al*., 2008; Doreau *et al*., 2009; Shingfield *et al*., 2011). Additionally, there has also been much interest in increasing the long-chain PUFA (LCPUFA; C20+) content of ruminant meat due to their beneficial health properties. Ruminants are able to undergo fatty acid chain elongation in their muscle tissue, meaning that for example *n*-3 PUFA may be converted to 20:5*n*-3 and subsequently to 22:6*n*-3 in the liver. Nonetheless, the process is inefficient, with some hypothesis suggesting that the initial conversion of 18:3*n*-3 to 18:4*n*-3 may be a rate-limiting step (Cleveland *et al*., 2012). As such, supplementation of ruminant diets with 18:4*n*-3 has been suggested as a potential way of circumventing the rate-limiting step and improving the production of LCPUFA in the muscle. Echium oil, derived from *Echium* spp. (plant rich in 18:4*n*-3), has been suggested as a potentially beneficial dietary supplement. However, two recent publications suggest that 18:4*n*-3 is largely biohydrogenated in the presence of rumen microbes *in vitro* (Alves *et al*., 2012; Maia *et al*., 2012), suggesting that little 18:4*n*-3 reaches the muscle or liver for enhancement of chain elongation *in vivo*.

While the effects of flax oil on the rumen fatty acids are well characterized, attempts to investigate the underlying rumen microbiome have used previously available profiling technology, and in this study we used next-generation sequencing to characterize the microbiome. In addition, we assessed the effect of supplementation of 18:4*n*-3 rich echium oil on the rumen lipidome and microbiome in order to understand whether the same levels of 18:4*n*-3 biohydrogenation are actually seen *in vivo* compared with the *in vitro* data, and to prospect the underlying changes in the rumen microbiome in detail. In-depth understanding of the rumen lipidome and microbiome are essential for increasing our fundamental understanding of rumen lipid metabolism.

## Results

### Diet composition

Dry matter (DM), water-soluble carbohydrate (WSC), total nitrogen, acid detergent fibre (ADF), neutral detergent fibre (NDF), ammonia-N and pH composition of the diet were identical. Fatty acid composition of the following diets – grass silage/sugar beet pulp only (GS), GS supplemented either with flax oil (GSF) and GS supplemented either with echium oil (GSE) – were similar with respect to 12:0, 14:0, 18:1 *trans*-11 and LCPUFA (Table 1). GSE and GSF had higher levels of 16:0, 18:0, 18:1 *trans*-10, 18:2*n*-6, 18:3*n*-3 and total fatty acids than the GS diet (Table 1). GSE also had higher 18:4*n*-3 than GS and GSF (Table 1).

**Table 1:** Chemical composition and fatty acid profile of the experimental diet and supplemented oils (g kg^−1^ DM).^a^

	Diets		
	GS	Flax oil	Echium oil
Dry matter (DM)	603	N/A	N/A
Water-soluble carbohydrate (WSC)	197	N/A	N/A
Total nitrogen (N)	252	N/A	N/A
Acid-detergent fibre (ADF)	391	N/A	N/A
Neutral-detergent fibre (NDF)	707	N/A	N/A
Ammonia-N	1.15	N/A	N/A
pH	4.99	N/A	N/A
Fatty acid composition			
12:0	0.085	0.000	0.001
14:0	0.308	0.013	0.010
16:0	4.308	1.611	2.064
18:0	0.376	1.757	1.014
18:1 *trans*-10	0.009	0.003	0.003
18:1 *trans*-11	0.006	0.000	0.000
18:2*n*-6	4.913	4.762	4.487
18:3*n*-3	4.908	16.132	10.082
18:4*n*-3	0.047	0.052	4.527
LCPUFA (C20 and above)	0.289	0.021	0.030
Total fatty acids	18.390	32.110	31.110

a Values are means; *n* = 6.

GS diet, grass silage and sugar beet; LCPUFA, long-chain polyunsaturated fatty acids; N/A, not applicable.

### Dietary intake

DM, WSC, total nitrogen, ADF, NDF, dietary intake on all diets were similar (Table 2). Fatty acid intake post feeding on GS, GSF and GSE diets were similar with respect to 12:0, 14:0, 18:1 *trans*-11and LCPUFA (Table 2). Intakes of 16:0, 18:0, 18:1 *trans*-10, 18:2*n*-6, 18:3*n*-3 and total fatty acids on GSE and GSF diets were higher than when steers were fed the GS diet. GSE feeding also resulted in a higher intake of 18:4*n*-3 than GS and GSF feeding (Table 2).

**Table 2:** Nutrient intake (kg day^−1^) and fatty acid intake (g day^−1^) for steers fed grass and sugar beet (GS), and GS with the addition of flax (GSF) or echium oil (GSE).*

	Diets			SED	*P*
	GS	GSF	GSE		
Dry matter (DM)	7.61^a^	7.64^a^	7.60^a^	0.02	0.998
Water-soluble carbohydrate (WSC)	1.49^a^	1.49^a^	1.49^a^	0.00	0.999
Total nitrogen (N)	1.92^a^	1.92^a^	1.92^a^	0.00	0.999
Acid-detergent fibre (ADF)	2.97^a^	2.97^a^	2.97^a^	0.00	0.999
Neutral-detergent fibre (NDF)	5.37^a^	5.39^a^	5.36^a^	0.01	0.998
Fatty acids					
12:0	0.61^a^	0.65^a^	0.65^a^	0.03	0.350
14:0	2.34^a^	2.42^a^	2.44^a^	0.11	0.654
16:0	32.7^a^	45.0^b^	48.4^b^	1.97	< 0.001
18:0	2.86^a^	16.2^c^	10.6^b^	0.52	< 0.001
18:1 *trans*-10	0.07^a^	0.09^b^	0.09^b^	0.00	< 0.001
18:1 *trans*-11	0.05^a^	0.05^a^	0.05^a^	0.00	0.999
18:2*n*-6	37.3^a^	73.5^b^	71.4^b^	2.91	< 0.001
18:3*n*-3	37.3^a^	160^c^	114^b^	5.33	< 0.001
18:4*n*-3	0.36^a^	0.75^a^	34.7^b^	0.93	< 0.001
LCPUFA (C20 and above)	2.20^a^	2.36^a^	2.43^a^	0.11	0.127
Total fatty acids	140^a^	384^b^	380^b^	14.8	< 0.001

* Values are means; *n* ≤ 5.

Numbers with a different superscript vary significantly (*P* < 0.05) from each other. SED, standard deviation.

### Effects of flax and echium oil supplementation on the steer rumen lipidome

Steer 4 was unwell leading up to the sampling period on the GS diet (period 1); therefore, samples could not be taken, so *n* = 5 for this diet. Also steer 3 rumen microbiome pyrosequences were > 2500 reads (see following section), so all data with respect to this animal were removed, so *n* = 5 for the GSE diet also (period 1). Irrespective, GS, GSF and GSE diets did not cause any changes to branched and odd chain fatty acids (BOC), and 12:0, 18:2 *trans*-10 and *cis*-12 concentrations within the rumen (*P* > 0.05; Table 3). Nonetheless, all other fatty acids were significantly changed due to the diet offered (Table 3). Specifically, when comparing with the GS diet, the SFAs 16:0, 18:0 and 20:0 were higher in steers fed GSF and GSE (*P* < 0.05). The *trans*-monounsaturated fatty acids 18:1 *trans*-6, *trans*-7, *trans*-8, 18:1 *trans*-9, 18:1 *trans*-10, 18:1 *trans*-11, 18:1 *trans*-12, as well as total 18:1 *trans* monounsaturated fatty acids, were higher in the rumen of steers fed GSF and GSE compared with those fed GS diets (*P* < 0.05). The *cis* monounsaturated fatty acids 18:1 *cis*-9, 18:1 *cis*-11, 18:1 *cis*-12, 18:1 *cis*-13, as well as total 18:1 *cis* monounsaturated fatty acids, were higher in the rumen of steers fed GSF and GSE compared with those fed GS diets (*P* < 0.05). The CLAs 18:2 *cis*-9, *trans*-11, 18:2 *trans*-9, *trans*-12, 18:2 *cis*-9, *cis*-12, 18:2 *trans*-11, *trans*-13 and sum total CLAs were higher in the rumen of steers fed GSF and GSE compared with those fed GS diets (*P* < 0.05). The PUFAs 18:2*n*-6 and 18:3*n*-3 were higher in the rumen of steers fed GSF and GSE compared with those fed GS diets (*P* > 0.05). Stearidonic acid (18:4*n*-3) was higher in concentration within the rumen of steers fed GSE compared with GS- and GSF-fed steers (*P* < 0.05). In terms of any diet-induced changes on LCPUFA, 20:4, 22:5 and 22:6 were undetectable following feeding of all diets; nonetheless, 20:5 and sum LCPUFA were higher in the rumen of steers fed GSF and GSE compared with those fed GS diets (*P* < 0.05). Total fatty acids were also higher in concentration within the rumen of steers fed GSF and GSE compared with those fed GS diets (*P* < 0.05).

**Table 3:** Fatty acid profile (mg g^−1^ DM) of ruminal digesta from steers fed grass and sugar beet (GS), and GS with the addition of flax (GSF) or echium oil (GSE).*

Fatty acid	Diets			SED	*P*
	GS	GSF	GSE		
Branched and odd chain fatty acids (BOC)	1.437^a^	1.450^a^	1.547^a^	0.060	0.231
12:0	0.612^a^	0.651^a^	0.653^a^	0.03	0.350
14:0	0.312^a^	0.336^b^	0.334^b^	0.007	0.016
16:0	3.207^a^	4.251^b^	4.980^c^	0.140	< 0.001
18:0	4.193^a^	9.783^b^	11.061^b^	0.580	< 0.001
18:1 *trans*-6,-7,-8	0.024^a^	0.246^b^	0.253^b^	0.009	< 0.001
18:1 *trans*-9	0.019^a^	0.166^b^	0.217^c^	0.006	< 0.001
18:1 *trans*-10	0.030^a^	0.201^b^	0.246^c^	0.001	< 0.001
18:1 *trans*-11	0.555^a^	2.999^b^	4.797^c^	0.320	< 0.001
18:1 *trans*-12	0.034^a^	0.231^b^	0.291^c^	0.013	< 0.001
Sum 18:1 trans	0.850^a^	4.926^b^	6.832^c^	0.352	< 0.001
18:1 *cis*-9	0.606^a^	3.423^b^	3.419^b^	0.176	< 0.001
18:1 *cis*-11	0.089^a^	0.120^b^	0.221^c^	0.008	< 0.001
18:1 *cis*-12	0.010^a^	0.082^b^	0.051^c^	0.008	< 0.001
18:1 *cis*-13	0.011^a^	0.027^b^	0.030^b^	0.003	< 0.001
Sum 18:1 cis	0.126^a^	0.378^b^	0.388^b^	0.021	< 0.001
18:2 *cis*-9, *trans*-11	0.029^a^	0.274^b^	0.332^c^	0.022	< 0.001
18:2 *trans*-9, *trans*-12	0.009^a^	0.054^b^	0.029^c^	0.006	< 0.001
18:2 *cis*-9, *cis*-12	1.429^a^	2.162^b^	2.331^b^	0.238	0.015
18:2 *trans*-10, *cis*-12	0.018^a^	0.019^a^	0.021^a^	0.003	0.538
18:2 *trans-*11, *trans*-13	0.016^a^	0.124^b^	0.125^b^	0.011	< 0.001
Sum 18:2 Conjugated linoleic acid	0.081^a^	0.470^b^	0.579^c^	0.033	< 0.001
18:2*n*-6	1.429^a^	2.162^b^	2.331^b^	0.240	0.020
18:3*n*-3	1.104^a^	3.670^b^	2.970^b^	0.374	< 0.001
18:4*n*-3	0.049^a^	0.041^a^	1.261^b^	0.170	< 0.001
20:0	0.190^a^	0.248^b^	0.257^b^	0.011	< 0.001
20:4	ND	ND	ND	NA	NA
20:5	0.000^a^	0.008^b^	0.013^c^	0.000	< 0.001
22:5	ND	ND	ND	NA	NA
22:6	ND	ND	ND	NA	NA
Sum LCPUFA (C20 and above)	0.681^a^	0.921^b^	1.166^c^	0.082	0.001
Total fatty acids	13.72^a^	35.75^b^	38.97^b^	2.145	< 0.001

* Values are means; *n* ≥ 5.

Numbers with a different superscript vary significantly (*P* < 0.05) from each other. ND, not detectable; SED, standard deviation.

When comparing rumen fatty acids post GSF feeding with those present following GSE feeding 16:0, 18:1 *trans*-9, 18:1 *trans*-10, 18:1 *trans*-11, 18:1 *trans*-12, 18:1 *cis*-11, 18:2 *cis*-9, *trans*-11, 18:4*n*-3, 20:5 and total LCPUFA (C20 +) were higher in the rumen of steers fed GSE compared with the GSF diet (Table 3). Conversely, 18:1 *cis-*12, 18:2 *trans*-9, and *trans*-12 were lower in the rumen of steers fed GSE compared with the GSF diet (Table 3).

### Effects of flax and echium oil supplementation on the steer rumen microbiome

Only 2204 pyrosequences were obtained for one GSE-fed steer (steer 3, period 1), which was far lower than that obtained for the other samples; therefore, these pyrosequences and all other data relating to this steer were not analysed further. For the remaining samples, a total of 570 483 reads were obtained post-qiime analysis, with average sequence being 377 bp (Table 4). Shannon diversity-based rarefaction curves showed that sequence depth was reasonable for all samples (Fig. S1).

**Table 4:** Summary of pyrosequencing data of 16S rDNA 454 pyrosequences within the rumen of steers fed grass silage and sugar beet (GS), or GS supplemented with flax (GSF) or echium oil (GSE), pre- and post-qiime filtering

Total number of reads (pre-qiime analysis)	724 785
Total number of reads (post-qiime analysis)	570 483
Total reads for GS rumen samples	95 468
Average reads per sample for GS rumen samples	19 093 (1204)
Total reads for GSF rumen samples	287 647
Average reads per sample for GSF rumen samples	47 941 (5679)
Total reads for GSE rumen samples	187 368
Average reads per sample for GSE rumen samples	37 473 (3788)
Average sequence length (bp) + standard deviation	377 (61.1)
Domain: bacteria	100%
Total number of phyla	9
Total number of classes	30
Total number of genera	183
Average OTUs per sample for GS rumen samples	5095 (532)
Average OTUs per sample for GSF rumen samples	7567 (779)
Average OTUs per sample for GSE rumen samples	5972 (597)

Values in brackets are standard deviations.

**Figure 1:**
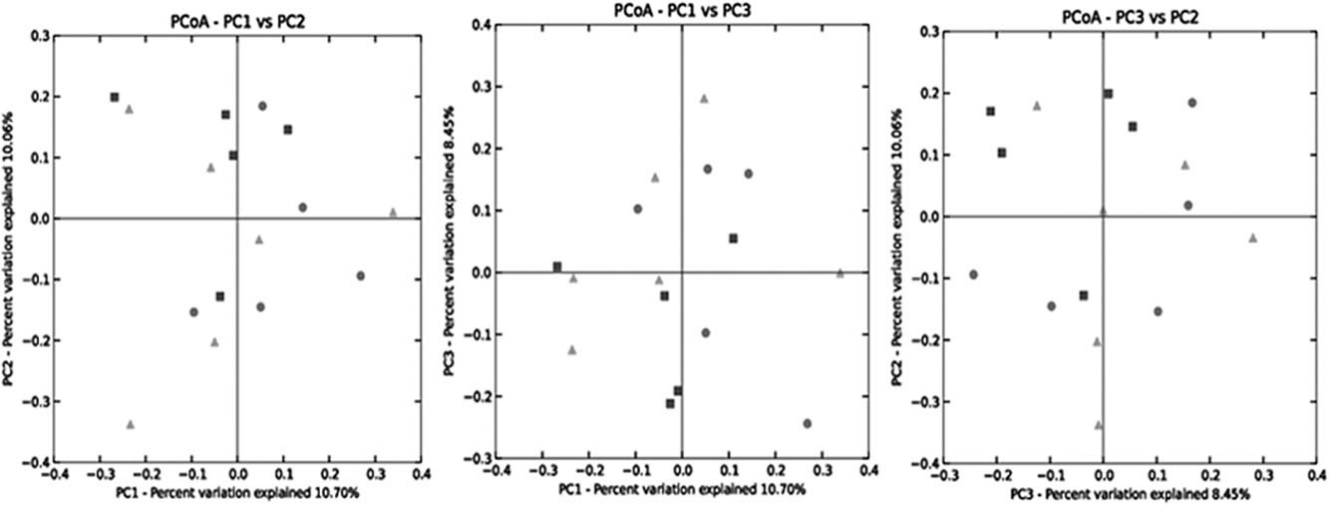
Unweighted UniFrac principal coordinates analysis (PCOA) of the rumen microbiome post-feeding steers grass silage/sugar beet (▪), or grass silage/sugar beet supplemented with flax oil (▴) or echium oil (•).

**Figure 2:**
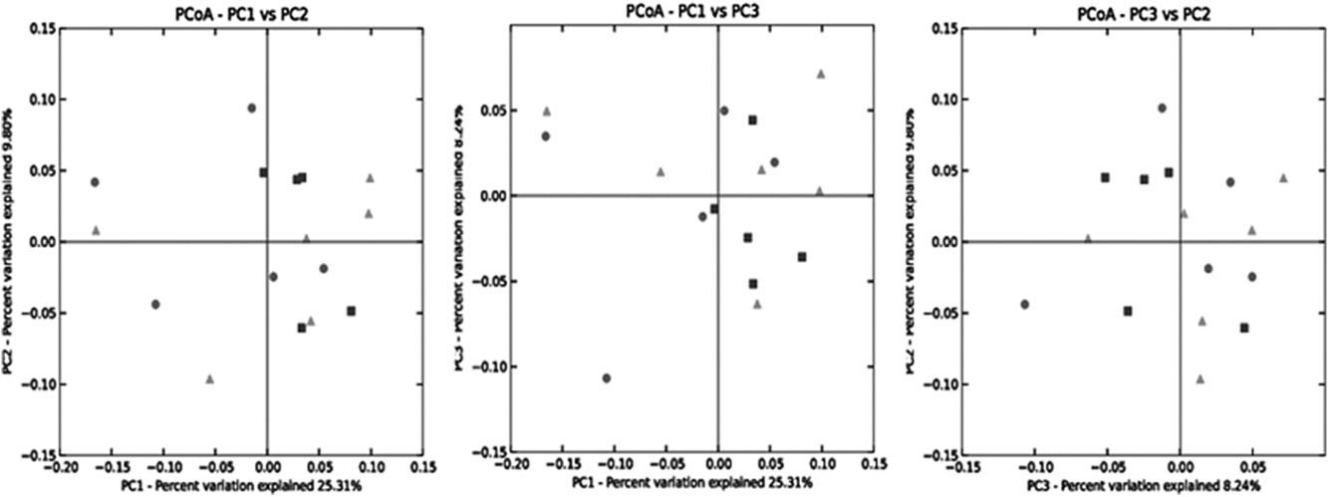
Weighted UniFrac principal coordinates analysis (PCOA) of the rumen microbiome post-feeding steers grass silage/sugar beet (▴), or grass silage/sugar beet supplemented with flax oil (▪) or echium oil (•).

Unweighted (Fig. 1) and weighted (Fig. 2) UniFrac principal coordinates analysis showed no overall unifying differences in the rumen microbiota following feeding of steers on any of the diets. Nonetheless, analysis of 454 pyrosequences at the phylum level showed that the *Actinobacteria* were higher in the rumen of steers fed GSE compared with GSF and GS (*P* < 0.05; Table S1). However, the other eight phyla did not differ dependent on steer diet (Table S1). Analysis of 454 pyrosequences at the class level showed no difference in bacterial diversity based on steer diet (Table S2). Analysis of 454 pyrosequences at the genus level showed that 24 out of the total of 183 genera differed in 16S rDNA concentration present within the rumen of the steers fed GSF compared with GS (*P* < 0.05; Table 5). When comparing GSF with GS diets, the rumen bacterial genera *Streptomyces*, *Olsonella*, *Bacteroidales*, and unclassified member of the *Bacteroidetes*, *Prevotellaceae*, *Prevotella*, *Anaerolinea*, *Fibrobacter*, *Clostridiales*, *Papillibacter*, *Ruminococcus*, *Eubacteriaceae*, *Clostridia*, *Erysipelotrichaceae*, Bacteria (other), *Victivallis*, *Firmicutes* and *Proteobacteria*, were higher in their 16S rDNA concentration within the rumen of steers fed GS compared with GSF diets (*P* < 0.05; Table 5). *Butyrivibrio*, *Howardella*, *Oribacterium*, *Pseudobutyrivibrio* and *Roseburia*, on the other hand, were lower in their 16S rDNA concentration within the rumen of steers fed GS compared with GSF diets (*P* < 0.05; Table 5). When comparing GSE with GS diets, the rumen bacterial unclassified genera within *Bacteroidales*, *Bacteroidetes*, *Anaerolinea*, *Lactobacillus*, *Eubacteriaceae*, *Victivallis* and *Proteobacteria* were higher in their 16S rDNA concentration within the rumen of steers fed GS compared with GSE diets, while the converse was true for *Succinovibrio* and *Roseburia* (*P* < 0.05; Table 5).

**Table 5:** Comparison of the bacteria (genus level) present within the rumen of steers fed grass silage and sugar beet (GS), or GS supplemented with flax (GSF) or echium oil (GSE)

Genus	Diet			SED	*P*
	GS	GSF	GSE		
*Olsonella*	0.461^b^	0.246^a^	0.427^b^	0.061	0.016
*Bacteroidales; other*	0.031^b^	0.013^a^	0.007^a^	0.005	0.007
*Bacteroidetes; other*	0.061^b^	0.013^a^	0.027^a^	0.012	0.010
*Prevotellaceae; other*	0.078^b^	0.029^a^	0.040^ab^	0.017	0.054
*Prevotella*	0.102^b^	0.033^a^	0.058^ab^	0.020	0.027
*Anaerolineaaceae; other*	0.020^b^	0.011^a^	0.011^a^	0.00	0.028
*Fibrobacter*	0.686^b^	0.218^a^	0.369^ab^	0.148	0.036
*Lactobacillales; other; other*	0.003^b^	0.002^ab^	0.000^a^	0.001	0.083
*Butyrivibrio*	8.552^a^	12.99^b^	8.407^a^	0.985	0.002
*Howardella*	0.004^a^	0.038^b^	0.012^a^	0.006	0.001
*Oribacterium*	0.102^a^	0.166^b^	0.076^a^	0.024	0.016
*Pseudobutyrivibrio*	2.526^a^	3.969^b^	2.602^a^	0.224	< 0.001
*Roseburia*	0.007^a^	0.038^c^	0.024^b^	0.005	0.001
*Clostridiales; other*	13.66^b^	12.02^a^	13.62^b^	0.547	0.028
*Papillibacter*	0.008^b^	0.002^a^	0.005^ab^	0.002	0.042
*Ruminococcus*	1.209^b^	0.461^a^	0.913^ab^	0.247	0.046
*Eubacteriaceae; other*	0.034^b^	0.0148^a^	0.016^a^	0.007	0.046
*Clostridia; other*	1.288^b^	0.773^a^	1.110^ab^	0.170	0.044
*Erysipelotrichaceae; other*	0.182^b^	0.071^a^	0.141^ab^	0.036	0.042
*Bacteria; other*	5.327^b^	3.505^a^	4.314^ab^	0.501	0.020
*Victivallis*	0.040^b^	0.017^a^	0.019^a^	0.007	0.016
*Firmicutes; other*	10.61^b^	8.154^a^	10.15^b^	0.283	< 0.01
*Succinivibrio*	0.001^a^	0.001^a^	0.010^b^	0.002	0.014
*Proteobacteria; other*	0.111^b^	0.090^ab^	0.063^a^	0.013	0.015

Only genera showing significant differences are shown in the table (*P* < 0.05) (data shown are % occurrences within the total reads). Numbers with a different superscript vary significantly (*P* < 0.05) from each other. SED, standard deviation.

**Figure 3:**
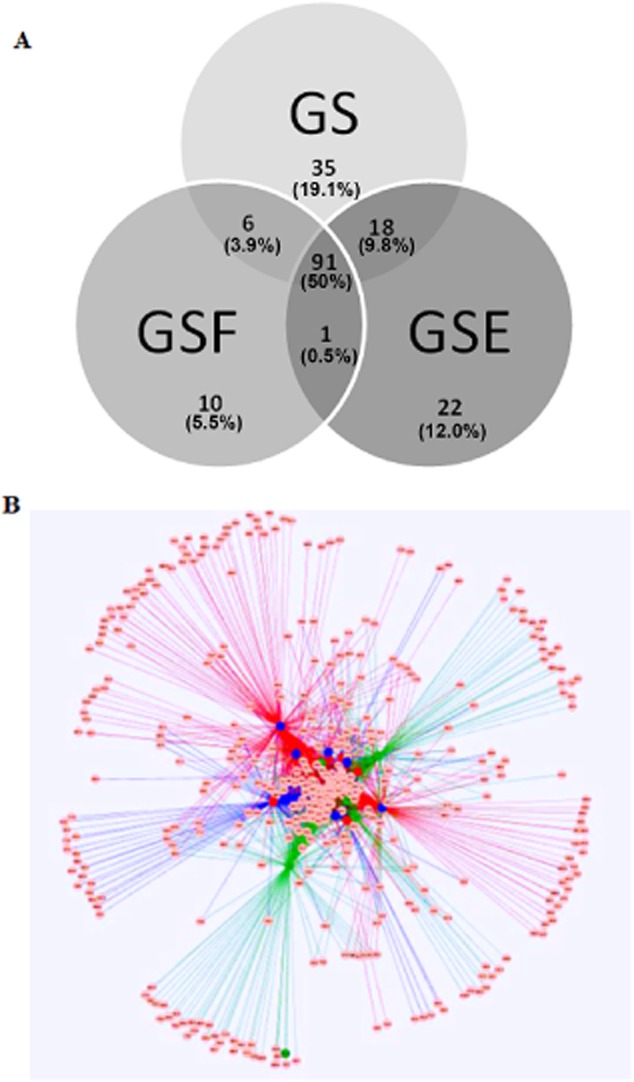
Venn diagram of the rumen core microbiome (found in each dietary intervention at least once) of steers fed grass silage (GS); red – GS and flax oil; blue – GS and echium oil, based on genus-level classification. Brackets show % genus overlap between diets and genera, which are core to all steers irrespective of diet (A). Spring-embedded weighted network map of microbiota with nodes representing operational taxonomic units (OTUs), and each line indicating that an OTU was identified in the same source (B). (Green – rumen samples from grass silage and sugar beet (GS)-fed steers; red – GS and flax oil; blue – GS and echium oil; network map was created based on steer so an evaluation of animal variation could be made).

The generated Venn diagram compiled at the genus level showed that 50% of the genera were core (found in microbiomes under all diets at least once/diet), 19.1%, 5.53% and 12.2% were unique (found at least once in the rumen microbiome of a steer fed a certain diet only) to the rumen microbiome of GS-, GSF- and GSE-fed steers, respectively, and 3.3%, 9.8% and 0.5% were shared between the microbiome of GS and GSF, GS and GSE, GSF and GSE respectively (Fig. 3A). When defining core as being genera present in all steers irrespective of diet, we found that 34 genera were core. Comparative analysis of this core microbiome (in all samples irrespective of diet) compared with the core microbiomes reported by Li and colleagues (2012) and Jami and Mizrahi (2012) showed that only six genera occurred in all samples within all studies, namely *Clostridium*, *Coprococcus*, *Eubacterium*, *Prevotella*, *Succiniclasticum* and members of the Ruminococcaceae (Table 6). When comparing our core microbiome (in all samples irrespective of diet) with that published by Jami and Mizrahi (2012), 13 of the same genera were found within samples from both studies (Table 6). In contrast, when comparing our core microbiome with that published by Li and colleagues (2012), 10 of the same genera were found (Table 6). Genus-level data for all steers on each diet showed a reasonable low level of variance with no steer being an obvious outlier (Tables S3–S5). An edge-weighted spring-embedded network map was generated from a heat map table, using calculated nodes and edges, in order to identify whether there were differences in the microbiome of the rumen of steers fed the differing diets at an operational taxonomic unit (OTU) level. The edge-weighted spring-embedded network map (Fig. 3B) revealed a core microbiome of 60.1% on an OTU basis; thus, 39.9% of OTUs were unique. There was also no significant difference for any obtained OTUs based on diet (data not shown).

**Table 6:** Comparison of the core microbiome (found within all our samples) within this study, and that reported by Li and colleagues (2012) and Jami and Mizrahi (2012) at the genus level

Genus (alphabetical order)	Our study	Li and colleagues (2012)	Jami and Mizrahi (2012)	Found in all three studies
*Acetivibrio*	+	+	−	N
*Adiercreutzia*	−	−	+	N
*Akkermansia*	−	+	−	N
*Aeromonadales*	−	−	+	N
*Alcaligenes*	+	−	−	N
*Alistipes*	−	+	−	N
*Anaerosporobacter*	+	−	−	N
*Anaerotruncus*	−	+	−	N
*Anaerovorax*	−	+	−	N
*Bacteria; other; other; other; other; other (unclassified)*	+	−	−	N
*Bacteroides*	+	+	−	N
*Barnesiella*	−	+	−	N
*Blautia*	+	−	+	N
*Bulleida*	−	+	+	N
*Butyricimonas*	−	+	−	N
*Butyrivibrio*	+	−	+	N
*Campylobacter*	−	+	−	N
*Cloacibacillus*	−	+	−	N
*Clostridium (including unclassified members)*	+	+	+	Y
*Comamonas*	−	+	−	N
*Coprococcus*	+	+	+	Y
*Coriobacteriaceae; other*	+	−	+	N
*Desulfovibrio*	−	+	+	N
*Dorea*	−	+	−	N
*Erysipelotrichaceae; other*	+	−	−	N
*Eubacterium*	+	+	+	Y
*Faecalibacterium*	−	+	−	N
*Fibrobacter*	+	+	−	N
*Firmicutes; other unclassified*	+	−	−	N
*Fusobacterium*	−	+	−	N
*F16*	−	−	+	N
*Guggenheimella*	−	+	−	N
*Lachnobacterium*	+	−	+	N
*Lachnospira*	−	−	+	N
*Lachnospiraceae (including unclassified members)*	+	−	+	N
*Microbacteriaceae*	+	−	−	N
*Mitzuokella*	−	−	+	N
*Moryella*	+	−	+	N
*Odoribacter*	−	+	−	N
*Olsonella*	+	−	−	N
*Oribacterium*	+	−	−	N
*Oscillospira*	−	−	+	N
*Paludibacter*	−	+	−	N
*Papillibacter*	−	+	−	N
*Parabacteroides*	−	+	−	N
*Paraprevotella*	−	+	−	N
*Phascolarctobacterium*	−	+	−	N
*Pigmentiphaga*	−	+	−	N
*Porphorymonas*	−	+	−	N
*Prevotella*	+	+	+	Y
*Pseudobutyrivibrio*	+	+	−	N
*Proteobacteria; other (unclassified)*	+	−	−	N
*p-75-a5*	−	−	+	N
*Rikenella*	−	+	−	N
*Robinsoniella*	−	+	−	N
*Roseburia*	−	+	+	N
*Ruminococcaceae (including unclassified members)*	+	+	+	Y
*Saccharofermentas*	+	−	−	N
*Selenomonas*	+	−	+	N
*Shuttleworthia*	−	−	+	N
*Sphingobacterium*	−	+	−	N
*Sporobacter*	−	+	−	N
*Streptococcus*	−	+	−	N
*Subdoligranulum*	−	+	−	N
*Succiniclasticum*	+	+	+	Y
*Succinivibrio*	−	+	−	N
*Tetrathiobacter*	−	+	−	N
*Treponema*	−	+	−	N
*Veillonella*	+	+	−	N
*Victivalis*	+	−	−	N
*YS2*	−	−	+	N

+, present; −, absent; Y, yes; N, no.

## Discussion

This study aimed to characterize changes in the rumen fatty acids and microbiome post-dietary supplementation of steers diets with flax and echium oil. Our data show that flax and echium oil supplementation of steer diets affects the rumen lipidome and underlying microbiome at the genus level.

Our depth of sequencing within this study is higher than those reported in many other 454 published data sets probing the rumen microbiome. For examples, Jami and colleagues (2013) obtained an average of 10 938 reads/sample, Fouts and colleagues (2012) obtained 23 493 reads, and Jami and Mizrahi (2012) reported an average 9587 reads/sample, whereas we obtained on average 34 835 reads/sample. From the reads, we discovered 9 phyla, 30 classes, 183 genera and an average of 6211 OTUs, which is similar to that obtained from other 454 based rumen microbiome data sets (Fouts *et al*., 2012; Jami and Mizrahi, 2012; Pope *et al*., 2012; Jami *et al*., 2013). In terms of the core (present in at least one steer under each diet) and unique microbiome (present only under one diet at least in one steer) within our data set, on an OTU basis we discovered that 60.1% were core to all diets, with the remainder being unique to individual steers. This is comparable to the 50% core rumen microbiome discovered by Jami and Mizrahi (2012) in the rumen of lactating steers based on OTUs. At the genus level, 91 genera were identified as core, with 92 being non-core genera, based on presence in at least one steer under each diet. When defining core as being found in all steers irrespective of diet, only 34 (19.7%) genera were core. Other studies reported 45 core genera (Li *et al*., 2012) and 32 core genera (Jami and Mizrahi, 2012), using this criteria; therefore, our data showing 34 core genera are similar at least in number. In terms of composition, the core microbiome (defined as present in all samples irrespective of diet) within our study, and those of Li and colleagues (2012) and Jami and Mizrahi (2012), shared 10 and 14 genera respectively. Both Li and colleagues (2012) and Jami and Mizrahi (2012) used different DNA extraction techniques and amplified different regions of the 16S rDNA gene compared with each other and with this study. This factor probably accounts for the low number of similar genera within all microbiomes compared, and it also highlights the challenges with comparing microbiome studies.

In terms of the diet effect on the rumen fatty acids, the GSF diet resulted in the intake of more 16:0 (× 1.4), 18:0 (× 5.7), 18:1 *trans*-10 (× 1.3), 18:2*n*-6 (× 2.0) and 18:3*n*-3 (× 4.3) compared with steers fed the GS diet. This may partially explain why these fatty acids were more concentrated within the rumen of steers fed GSF compared with GS. Greater supply of 18:3*n*-3 and 18:2*n*-6 resulted in the emergence of biohydrogenation intermediates, such as 18:1 *trans*-11 and many CLAs post feeding of GSF compared with the GS diet. The GSF diet resulted in higher concentrations of 18:3*n*-3 and 18:2*n*-6 within the rumen compared with GS-fed steers. It is difficult to compare our data on rumen lipidome changes with those that are published for flax oil supplementation due to the fact that previous studies have monitored the effects *in vitro* (Jouany *et al*., 2007) or have monitored flow of fatty acids to the animals' omasum or duodenum (Doreau *et al*., 2009; Shingfield *et al*., 2011; Sterk *et al*., 2012). Due to the fact that this study was focused on understanding the underlying microbiome related to the lipidome, these parameters were measured from the rumen within our study. Nonetheless, when comparing our data with those whereby omasal or duodenal flow was monitored, it is clear that we found the same trends (Doreau *et al*., 2009; Shingfield *et al*., 2011; Sterk *et al*., 2012). Specifically, the GSE diet resulted in higher rumen concentrations of all the fatty acids monitored, apart from BOC and 12:0, compared with those present in the rumen of steers fed the GS diet. It appears that much 18:4*n*-3, 18:3*n*-3 and 18:2*n*-6 were lost through the process of biohydrogenation, resulting in the emergence of biohydrogenation intermediates, such as 18:1 *trans*-11 and many CLAs post feeding of GSE compared with the GS diet. Irrespective, some 18:4*n*-3 remained unbiohydrogenated resulting in its higher concentrations within the rumen of GSE-fed steers compared with GS- and GSF-fed steers. The biohydrogenation of 18:4*n*-3 by the rumen microbiota has previously been shown *in vitro* (Alves *et al*., 2012; Maia *et al*., 2012), with similar effects on the lipidome as seen within our animal trial in terms of its rapid biohydrogenation. Alves and colleagues (2012), nonetheless, showed that 18:4*n*-3 biohydrogenation seems to follow an isomerization pattern with the migration of distinct double bonds shown to triene intermediates. In our study, we did not see these unique 18:3 intermediates likely due to our detection method, as Alves and colleagues (2012) used gas liquid chromatography-mass spectrophotometry to find these intermediates. When comparing results for rumen fatty acid concentration from steers-fed GSF as compared with those fed GSE, a higher accumulation of the biohydrogenation intermediates 18:1 *trans*-11 and 18:2 *cis*-9, *trans*-11, was seen in the rumen of steers fed GSE as compared with GSF diets; nonetheless, no significant differences in resultant 18:0 were seen. In a parallel study, we have also analysed the muscle lipidome from steers fed these three diets, and the data resemble the rumen fatty acid data in that the large reductions in PUFA and increases in biohydrogenation intermediates meant that significant increases in beneficial fatty acids for human health were not seen in the muscle of animals fed GSF and GSE (as yet unpublished data).

Using massive parallel sequencing, we found that GSF diet reduced the 16S rDNA abundance of the genera *Streptomyces*, *Olsonella*, *Bacteroidales*, *Bacteroidetes*, *Prevotellaceae*, *Prevotella*, *Anaerolinea*, *Fibrobacter*, *Clostridiales*, *Papillibacter*, *Ruminococcus*, *Eubacteriaceae*, *Clostridia*, *Erysipelotrichaceae*, *Bacteria* (other), *Victivallis*, *Firmicutes* and *Proteobacteria*, whereas *Butyrivibrio*, *Howardella*, *Oribacterium*, *Pseudobutyrivibrio* and *Roseburia* were higher in 16S rDNA abundance compared with the rumen microbiome of steers fed the GS diet. Yang and colleagues (2009) used quantitative PCR to look at changes in *Butyrivibrio fibrisolvens*, *Ruminococcus albus*, *R. flavefaciens* and *Fibrobacter succinogenes* in the rumen of dairy steers post feeding of flax oil at 4% DM intake of a basal diet composed of 60:40 forage : concentrate, and found that 16S rDNA abundances of all four species were reduced compared with their abundance on the basal diet only. We also noted that the 16S rDNA abundances of the genera *Fibrobacter* and *Ruminococcus* were reduced in comparison to abundances seen in the rumen of GS-fed steers. Nonetheless, within this study, we note an increase in the genus Butyrivibrio within the rumen of steers fed GSF compared with GS feeding. The bacterial genera *Butyrivibrio*, *Pseudobutyrivibrio* and *Roseburia* have been implicated in the process of biohydrogenation and may account for the high level of biohydrogenation intermediates seen when steers were fed GSF (Devillard *et al*., 2007; Paillard *et al*., 2007; Boeckaert *et al*., 2008). The role of genus Butyrivibrio in biohydrogenation is, however, unclear from data obtained within many studies (Kim *et al*., 2008; Huws *et al*., 2011; Toral *et al*., 2012), and as such the role of this genus within the rumen remains uncertain, although our sequencing data in this instance suggest a potential role in the biohydrogenation of flax oil PUFAs.

When the steers were fed the GSE diet, we found that the 16S rDNA concentration for the rumen bacterial genera *Bacteroidales*, *Bacteroidetes*, *Anaerolinea*, *Lactobacillus*, *Eubacteriaceae*, *Victivallis* and *Proteobacteria* were reduced in the rumen compared with steers fed GS. The rumen bacterial genera *Succinovibrio* and *Roseburia* were the only genera that were higher in their 16S rDNA concentration within the rumen of steers fed GSE compared with GS diets. Maia and colleagues (2007) demonstrated that the bacteria likely biohydrogenate due to the toxic nature of the double bond; therefore, it is perhaps unsurprising that when GSF and GSE are fed, many bacterial genera are reduced in terms of their 16S rDNA abundance. This is more prominent when echium oil was fed due to the increased unsaturated nature of 18:4*n*-3.

In summary, in this study, we characterized the rumen lipidome and microbiome upon feeding flax and echium oil supplements to cattle. We showed that feeding flax and echium oil supplementation changed the rumen lipidome substantially, compared with GS-fed steers. Furthermore, substantial conversion of 18:4*n*-3 was evident within the fatty acid profiles of steers fed a diet supplemented with echium oil. Concomitantly, we demonstrate that *Butyrivibrio*, *Howardella*, *Oribacterium*, *Pseudobutyrivibrio* and *Roseburia* 16S rDNA were higher within the rumen microbiome of GSF, and higher *Succinovibrio* and *Roseburia* 16S rDNA sequences were found within the microbiome of GSE-fed steers. The potential involvement of these bacteria in biohydrogenation requires further investigation. In-depth understanding of the rumen lipidome and microbiome is essential for increasing our fundamental understanding of rumen lipid metabolism.

## Experimental procedures

### Animals and allocation to treatment

The experiment was conducted under the authorities of the UK Animal (Scientific Procedures) Act (1986). Six Hereford × Friesian (*Bos taurus*) steers (mean live weight 534.6 kg) prepared with ruminal cannulae and simple ‘T'-piece cannulae in the proximal duodenum (immediately post-pylorus and pre-common bile duct) (Jarret, 1948) were offered grass silage and sugar beet pulp (GS diet) or grass silage/sugar beet (*Beta vulgaris*) pulp supplemented either with flax oil (GSF diet) or echium oil (GSE diet; echium oil derived from *Echium plantagineum*) (both at 3% kg^−1^ silage DM). The total daily allowance was set at 14 g DM kg^−1^ live weight to ensure complete daily consumption with a forage : concentrate ratio of 60:40 (DM basis). Steers were housed in individual pens, and transferred to stalls for each measurement period. The building was well ventilated, with steers having free access to fresh water and mineral blocks (Baby Red Rockies, Tithebarn, Winsford, Cheshire, UK; composed of 380 g kg^−1^ Na, 5000 mg kg^−1^ Mg, 1500 mg kg^−1^ Fe, 300 mg kg^−1^ Cu, 300 mg kg^−1^ Zn, 200 mg kg^−1^ Mn, 150 mg kg^−1^ I, 50 mg kg^−1^ Co and 10 mg kg^−1^ Se). The experiment consisted of a three-period replicated Latin square design with 21-day periods. Each 21-day (d) period consisted of 20 days adaptation to the experimental diets and 1 day for sample collection. Steers received their daily allocations in two equal meals at 09:00 and 16:00.

### Sample preparation and chemical analysis

Separate samples of silage were taken daily, whereas a sample of concentrate, flax and echium oil was taken for each period. Subsamples of silage and concentrate were freeze-dried, ground and retained at −20°C for chemical analysis. Rumen fluid was taken on day 21 of each period and strained through two layers of muslin before contents were frozen at −20°C, and subsequently freeze-dried and ground. At the same time, a separate sample (450 g) of strained solids was taken and combined with 100 ml of strained rumen liquor, and freeze-dried (DM recorded), ground and retained frozen at −20°C for fatty acid analysis (Huws *et al*., 2011). DM, WSC, NDF, ADF, total nitrogen (N) and fatty acid composition of collected samples were analysed, as by Lee and colleagues (2002).

### DNA extraction

Genomic DNA was extracted from rumen fluid (10 mg DM) using the BIO101 FastDNA® SPIN Kit for Soil (Qbiogene, Cambridge, UK) in conjunction with a FastPrep® cell disrupter instrument (Bio101, ThermoSavant, Qbiogene) according to the manufacturer's instructions with the exception that the samples were processed for 3 × 30 s at speed 6.0 in the FastPrep instrument. Previous optimization studies have shown that this kit and these parameters result in enhanced extraction of DNA from Gram + ve rumen bacteria, and therefore a realistic representation of the rumen microbiome (data not shown). DNA was quantified and quality-assured using the Epoch microplate spectrophotometer (Biotek, Bedfordshire, UK).

### 16S rDNA 454 pyrosequencing

Amplicons of the V6–V8 variable region of the bacterial 16S rDNA gene were generated in triplicate per DNA sample by PCR using the primers F968 (5′ tagged with Roche B adaptor) and R1401 (5′ tagged with the Roche A adaptor and MID barcode tags specific for each sample as suggested by Roche) as described by Huws and colleagues (2011), except that 30 cycles of amplification were used. All PCR products were initially verified by electrophoretic fractionation on a 1.0% agarose gel for 1 h, 120 V and 80 MA in 1% TAE (Tris base, acetic acid and EDTA) buffer before pooling of triplicate amplifications. The pooled PCR products (30 μl each sample) were subsequently run on a 2.0% agarose gel for 2 h, 120 V and 80 MA in 1% TAE buffer before bands were viewed and cut on a dark reader transilluminator (Clare Chemical Research, Colorado, USA). Amplicons were retrieved from cut bands using the Isolate II PCR and Gel Kit (Bioline, London, UK). Purified amplicons were verified and quantified using the Agilent High Sensitivity Assay Kit (Agilent Technologies, California, USA) prior to pyrosequencing using Titanium chemistry on a Roche GS-FLX 454 sequencer (Roche Diagnostics, West Sussex, UK) using the manufacturer's guidelines. These sequence data have been submitted to the short read archive under accession number SRP036181.

### Statistical analysis

Pyrosequencing data were analysed using qiime version 2.1 (Caporaso *et al*., 2010) in the Bio-Linux 7 environment (Field *et al*., 2006). Reads were split into samples through their barcodes, and reads were then quality-filtered following the default qiime parameters, except for a minimum quality score of 25, minimum number of mismatches in primers of zero, maximum homopolymer run of six and maximum number of ambiguous bases of zero. Taxonomic classifications were assigned against the Ribosomal Database Project database based on 97% similarity (Cole *et al*., 2009) using uclust (Edgar, 2010). The resulting taxonomic classifications and their relevant abundance in each sample were exported as a biological observation matrix, and further analysis was completed in Microsoft Excel 2010. A Venn diagram was constructed in Microsoft Excel from exported genus-level data. The OTU edge-weighted spring-embedded network map was compiled using nodes and edges generated from a heat map table and using Cytoscape (Shannon *et al*., 2003). Relative OTU abundance differences seen within the generated heat map were also extracted for statistical analysis. Fatty acid data and taxonomical tables on phyla, family, genus and OTU level were subjected to analysis of variance (ANOVA) with diet as the treatment effect and blocking according to period + animal using GenStat (Payne *et al*., 2007). Missing values were noted by an asterisk in the Excel sheet and likely estimated values calculated by the GenStat software for incorporation into the ANOVA. This allowed statistical analysis of a full Latin square design.

## Conflict of interest

None of the authors have any conflict of interest to declare.

## Supporting Information

**Fig. S1.** Shannon diversity rarefaction indices for sequences obtained from cows fed grass silage/sugar beet (GS), GS and flax oil (GSF) and GS and echium oil (GSE). The lines all overlay each other for each sample.

**Table S1.** Comparison of the bacteria (Phylum level) present within the rumen of steers fed grass silage and sugar beet (GS), or GS supplemented with flax (GSF) or echium oil (GSE) (Data shown are % occurrences within the total reads).

**Table S2.** Comparison of the bacteria (Class level) present within the rumen of steers fed grass silage and sugar beet (GS), or GS supplemented with flax (GSF) or echium oil (GSE) (Data shown are % occurrences within the total reads).

**Table S3.** Comparison of the bacteria (Genus level) present within the rumen of steers fed grass silage and sugar beet (GS diet) only. Data shown are % occurrences within the total reads. Only sequences occurring above 0.001% of total read abundance are shown.

**Table S4.** Comparison of the bacteria (Genus level) present within the rumen of steers fed grass silage and sugar beet and flax oil (GSF diet). Data shown are % occurrences within the total reads. Only sequences occurring above 0.001% of total read abundance are shown.

**Table S5.** Comparison of the bacteria (Genus level) present within the rumen of steers fed grass silage/sugar beet and echium oil. Data shown are % occurrences within the total reads. Only sequences occurring above 0.001% of total read abundance are shown.
