# Supplementary figures and images for: Characterization of the rumen lipidome and microbiome of steers fed a diet supplemented with flax and echium oil
Source: Microb Biotechnol. 2014 Sep 16;8(2):331–41. doi: 10.1111/1751-7915.12164 (PMC4353346; doi:10.1111/1751-7915.12164)

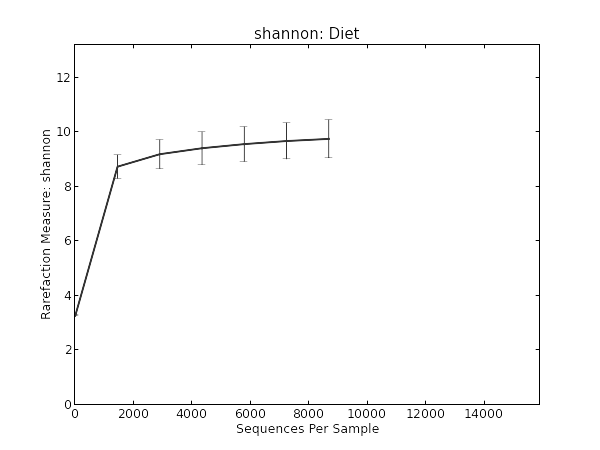

Supplement: Supplementary file 1 [file mbt20008-0331-sd1.tif]
