# Supplementary material for: Characterization of the rumen lipidome and microbiome of steers fed a diet supplemented with flax and echium oil
Source: Microb Biotechnol. 2014 Sep 16;8(2):331–41. doi: 10.1111/1751-7915.12164 (PMC4353346; doi:10.1111/1751-7915.12164)
Supplement: Supplementary file 3 [file mbt20008-0331-sd3.docx]

**Supplementary Table 2** Comparison of the bacteria (Class level) present within the rumen of steers fed grass silage and sugar beet (GS), or GS supplemented with flax (GSF) or echium oil (GSE) (Data shown are % occurrences within the total reads).

|  |  | Diet |  |  |  |
| --- | --- | --- | --- | --- | --- |
| Class | GS | GSF | GSE | SED | *P* |
| Actinobacteria | 0.705^a^ | 0.943^a^ | 1.116^a^ | 0.218 | 0.246 |
| Alphaproteobacteria | 0.022^a^ | 0.022^a^ | 0.073^a^ | 0.035 | 0.311 |
| Anaerolinea | 0.008^a^ | 0.016^a^ | 0.019^a^ | 0.005 | 0.166 |
| Bacilli | 0.041^a^ | 0.044^a^ | 0.069^a^ | 0.019 | 0.350 |
| Bacteria; other | 3.876^a^ | 4.298^a^ | 4.842^a^ | 0.678 | 0.416 |
| Bacteroidetes; other | 0.027^a^ | 0.039^a^ | 0.034^a^ | 0.013 | 0.683 |
| Bacteroidia | 0.145^a^ | 0.238^a^ | 0.233^a^ | 0.077 | 0.454 |
| Betaproteobacteria | 0.060^a^ | 0.064^a^ | 0.032^a^ | 0.014 | 0.128 |
| Chlamydiae | 0.010^a^ | 0.010^a^ | 0.010^a^ | 0.005 | 0.967 |
| Chloroflexi; other | 0.000^a^ | 0.000^a^ | 0.002^a^ | 0.002 | 0.282 |
| Clostridia | 84.22^a^ | 82.91^a^ | 81.31^a^ | 1.685 | 0.297 |
| Deltaproteobacteria | 0.079^a^ | 0.090^a^ | 0.118^a^ | 0.017 | 0.131 |
| Elusimicrobia | 0.001^a^ | 0.000^a^ | 0.000^a^ | 0.000 | 0.205 |
| Epsilonproteobacteria | 0.000^a^ | 0.000^a^ | 0.000^a^ | 0.000 | 0.124 |
| Erysipelotrichia | 0. 140^a^ | 0.211^a^ | 0.297^a^ | 0.119 | 0.466 |
| Fibrobacteria | 0.384^a^ | 0.518^a^ | 0.371^a^ | 0.166 | 0.637 |
| Firmicutes; other | 9.282^a^ | 9.357^a^ | 9.948^a^ | 0.319 | 0.152 |
| Gammaproteobacteria | 0.035^a^ | 0.024^a^ | 0.048^a^ | 0.010 | 0.152 |
| Lentisphaeria | 0.021^a^ | 0.028^a^ | 0.028^a^ | 0.007 | 0.525 |
| Mollicutes | 0.000^a^ | 0.000^a^ | 0.001^a^ | 0.001 | 0.839 |
| Negativicutes | 0.412^a^ | 0.495^a^ | 0.660^a^ | 0.129 | 0.227 |
| Opitutae | 0.008^a^ | 0.009^a^ | 0.010^a^ | 0.005 | 0.876 |
| Proteobacteria; other | 0.082^a^ | 0.107^a^ | 0.075^a^ | 0.015 | 0.152 |
| Sphingobacteria | 0.000^a^ | 0.000^a^ | 0.000^a^ | 0.000 | 0.282 |
| Spirochaetes | 0.004^a^ | 0.008^a^ | 0.012^a^ | 0.005 | 0.372 |
| Synergistia | 0.000^a^ | 0.000^a^ | 0.000^a^ | 0.000 | 0.364 |
| Unclassified; other | 0.011^a^ | 0.019^a^ | 0.012^a^ | 0.007 | 0.560 |
| Verrucomicrobia; other | 0.066^a^ | 0.047^a^ | 0.055^a^ | 0.010 | 0.259 |
| Verrucomicrobia; subdivision 5 | 0.357^a^ | 0.503^a^ | 0.619^a^ | 0.108 | 0.128 |
| Verrucomicrobiae | 0.000^a^ | 0.000^a^ | 0.004^a^ | 0.003 | 0.341 |

Data with different superscripts differed significantly (*P*<0.05)
