# Supplementary material for: Characterization of the rumen lipidome and microbiome of steers fed a diet supplemented with flax and echium oil
Source: Microb Biotechnol. 2014 Sep 16;8(2):331–41. doi: 10.1111/1751-7915.12164 (PMC4353346; doi:10.1111/1751-7915.12164)
Supplement: Supplementary file 4 [file mbt20008-0331-sd4.docx]

**Table 3 Supplementary** Comparison of the bacteria (Genus level) present within the rumen of steers fed grass silage and sugar beet (GS diet) only. Data shown are % occurrences within the total reads. Only sequences occurring above 0.001% of total read abundance are shown.

|  |  | Steer number | | | | Average | | | SED | |  |
| --- | --- | --- | --- | --- | --- | --- | --- | --- | --- | --- | --- |
|  | 1 | 2 | 3 | 5 | 6 | |  |  | |  | |

| *Corynebacterium* | 0.003 | 0.038 | 0.000 | 0.001 | 0.016 | 0.010 | 0.016 |
| --- | --- | --- | --- | --- | --- | --- | --- |
| *Microbacterium* | 0.000 | 0.000 | 0.020 | 0.006 | 0.000 | 0.004 | 0.009 |
| *Microbacteriaceae;Other* | 0.005 | 0.007 | 0.020 | 0.011 | 0.008 | 0.009 | 0.006 |
| *Nocardiaceae;Other* | 0.000 | 0.002 | 0.020 | 0.002 | 0.000 | 0.004 | 0.008 |
| *Marmoricola* | 0.000 | 0.001 | 0.000 | 0.000 | 0.008 | 0.002 | 0.003 |
| *Actinomycetales;Other;Other* | 0.002 | 0.028 | 0.000 | 0.008 | 0.008 | 0.008 | 0.011 |
| *Propionibacteriaceae;Other* | 0.000 | 0.000 | 0.000 | 0.001 | 0.032 | 0.005 | 0.014 |
| *Atopobium* | 0.010 | 0.043 | 0.020 | 0.010 | 0.032 | 0.019 | 0.014 |
| *Eggerthella* | 0.007 | 0.016 | 0.000 | 0.008 | 0.016 | 0.008 | 0.007 |
| *Olsenella* | 0.176 | 0.933 | 0.975 | 0.286 | 0.845 | 0.536 | 0.381 |
| *Coriobacteriaceae;Other* | 0.225 | 0.723 | 0.597 | 0.291 | 0.494 | 0.388 | 0.208 |
| *Slackia* | 0.005 | 0.085 | 0.000 | 0.038 | 0.064 | 0.032 | 0.037 |
| *Actinobacteria;Other;Other;Other* | 0.000 | 0.016 | 0.020 | 0.000 | 0.000 | 0.006 | 0.010 |
| *Bacteroidales;Other;Other* | 0.047 | 0.250 | 0.199 | 0.029 | 0.072 | 0.100 | 0.099 |
| *Porphyromonadaceae;Other* | 0.024 | 0.047 | 0.040 | 0.010 | 0.032 | 0.026 | 0.014 |
| *Howardella* | 0.000 | 0.007 | 0.000 | 0.001 | 0.008 | 0.003 | 0.004 |
| *Prevotellaceae;Other* | 0.030 | 0.162 | 0.040 | 0.023 | 0.128 | 0.064 | 0.064 |
| *Prevotella* | 0.042 | 0.192 | 0.159 | 0.023 | 0.120 | 0.089 | 0.073 |
| *Bacteroidetes;Other;Other;Other;Other* | 0.024 | 0.109 | 0.080 | 0.027 | 0.072 | 0.052 | 0.036 |
| *Parachlamydiaceae;Other* | 0.005 | 0.001 | 0.000 | 0.002 | 0.000 | 0.001 | 0.002 |
| *Parachlamydia* | 0.012 | 0.004 | 0.020 | 0.005 | 0.000 | 0.007 | 0.008 |
| *Anaerolineaceae;Other* | 0.023 | 0.028 | 0.020 | 0.007 | 0.024 | 0.017 | 0.008 |
| *Fibrobacter* | 0.304 | 1.655 | 0.756 | 0.246 | 0.462 | 0.571 | 0.577 |
| *Bacillales;Other;Other* | 0.002 | 0.000 | 0.000 | 0.005 | 0.000 | 0.001 | 0.002 |
| *Cohnella* | 0.000 | 0.001 | 0.000 | 0.000 | 0.008 | 0.002 | 0.003 |
| *Paenibacillus* | 0.002 | 0.002 | 0.020 | 0.007 | 0.016 | 0.008 | 0.008 |
| *Leuconostoc* | 0.000 | 0.002 | 0.000 | 0.007 | 0.000 | 0.002 | 0.003 |
| *Weissella* | 0.007 | 0.016 | 0.040 | 0.016 | 0.016 | 0.016 | 0.012 |
| *Lactobacillales;Other;Other* | 0.000 | 0.000 | 0.020 | 0.002 | 0.000 | 0.004 | 0.009 |
| *Streptococcaceae;Streptococcus* | 0.002 | 0.014 | 0.020 | 0.004 | 0.008 | 0.008 | 0.007 |
| *Anaerosporobacter* | 0.019 | 0.028 | 0.060 | 0.022 | 0.032 | 0.027 | 0.016 |
| *Mogibacterium* | 0.122 | 0.052 | 0.080 | 0.108 | 0.056 | 0.070 | 0.031 |
| *Anaerofustis* | 0.009 | 0.005 | 0.020 | 0.002 | 0.000 | 0.006 | 0.008 |
| *Eubacterium* | 0.229 | 0.093 | 0.020 | 0.217 | 0.223 | 0.130 | 0.095 |
| *Eubacteriaceae;Other* | 0.014 | 0.025 | 0.040 | 0.021 | 0.064 | 0.027 | 0.020 |
| *Blautia* | 0.178 | 0.084 | 0.100 | 0.223 | 0.199 | 0.131 | 0.062 |
| *Butyrivibrio* | 9.154 | 7.683 | 6.827 | 9.622 | 6.750 | 6.673 | 1.323 |
| *Clostridium XlVb* | 0.016 | 0.004 | 0.000 | 0.020 | 0.048 | 0.015 | 0.019 |
| *Coprococcus* | 0.105 | 0.110 | 0.119 | 0.170 | 0.167 | 0.112 | 0.032 |
| *Lachnobacterium* | 0.028 | 0.015 | 0.080 | 0.012 | 0.016 | 0.025 | 0.028 |
| *Lachnospiracea_incertae_sedis* | 1.042 | 0.360 | 0.896 | 0.824 | 0.598 | 0.620 | 0.268 |
| *Lactonifactor* | 0.002 | 0.001 | 0.020 | 0.003 | 0.000 | 0.004 | 0.008 |
| *Marvinbryantia* | 0.000 | 0.000 | 0.020 | 0.000 | 0.000 | 0.003 | 0.009 |
| *Moryella* | 0.723 | 0.364 | 0.677 | 0.723 | 0.566 | 0.509 | 0.152 |
| *Oribacterium* | 0.108 | 0.071 | 0.080 | 0.092 | 0.167 | 0.086 | 0.038 |
| *Lachnospiraceae;Other* | 51.154 | 33.297 | 35.888 | 49.154 | 44.625 | 35.686 | 7.931 |
| *Pseudobutyrivibrio* | 2.600 | 1.946 | 2.926 | 2.692 | 2.295 | 2.076 | 0.380 |
| *Robinsoniella* | 0.000 | 0.006 | 0.000 | 0.001 | 0.000 | 0.001 | 0.003 |
| *Roseburia* | 0.017 | 0.010 | 0.000 | 0.014 | 0.008 | 0.008 | 0.007 |
| *Shuttleworthia* | 0.005 | 0.002 | 0.000 | 0.004 | 0.000 | 0.002 | 0.002 |
| *Syntrophococcus* | 0.010 | 0.016 | 0.020 | 0.005 | 0.000 | 0.009 | 0.008 |
| *Clostridiales;Other;Other* | 13.979 | 14.515 | 12.759 | 14.027 | 12.280 | 11.260 | 0.945 |
| *Acetivibrio* | 0.059 | 0.120 | 0.080 | 0.077 | 0.064 | 0.067 | 0.024 |
| *Clostridium IV* | 0.381 | 1.023 | 1.274 | 0.491 | 1.474 | 0.774 | 0.479 |
| *Flavonifractor* | 0.002 | 0.015 | 0.000 | 0.002 | 0.000 | 0.003 | 0.006 |
| *Oscillibacter* | 0.016 | 0.041 | 0.040 | 0.022 | 0.024 | 0.024 | 0.011 |
| *Ruminococcaceae;Other* | 1.910 | 6.807 | 6.967 | 2.941 | 4.638 | 3.877 | 2.261 |
| *Papillibacter* | 0.002 | 0.014 | 0.020 | 0.007 | 0.000 | 0.007 | 0.008 |
| *Ruminococcus* | 0.829 | 1.299 | 2.150 | 0.514 | 1.387 | 1.030 | 0.622 |
| *Saccharofermentans* | 0.885 | 3.277 | 3.205 | 0.957 | 2.343 | 1.778 | 1.167 |
| *Pelospora* | 0.002 | 0.009 | 0.020 | 0.009 | 0.016 | 0.009 | 0.007 |
| *Clostridia;Other;Other;Other* | 0.761 | 1.793 | 1.393 | 0.948 | 1.466 | 1.060 | 0.415 |
| *Bulleidia* | 0.058 | 0.109 | 0.020 | 0.028 | 0.151 | 0.061 | 0.056 |
| *Catenibacterium* | 0.016 | 0.047 | 0.080 | 0.041 | 0.016 | 0.033 | 0.026 |
| *Erysipelotrichaceae;Other* | 0.079 | 0.292 | 0.259 | 0.093 | 0.183 | 0.151 | 0.096 |
| *Succiniclasticum* | 0.192 | 0.857 | 0.717 | 0.244 | 0.590 | 0.433 | 0.292 |
| *Anaerovibrio* | 0.000 | 0.006 | 0.000 | 0.000 | 0.000 | 0.001 | 0.003 |
| *Veillonellaceae;Other* | 0.047 | 0.254 | 0.159 | 0.049 | 0.112 | 0.104 | 0.086 |
| *Schwartzia* | 0.000 | 0.012 | 0.040 | 0.001 | 0.000 | 0.009 | 0.017 |
| *Selenomonas* | 0.012 | 0.080 | 0.080 | 0.026 | 0.024 | 0.037 | 0.033 |
| *Firmicutes;Other;Other;Other;Other* | 9.700 | 11.714 | 11.525 | 10.024 | 10.806 | 8.961 | 0.889 |
| *Victivallis* | 0.023 | 0.052 | 0.060 | 0.020 | 0.040 | 0.032 | 0.017 |
| *Bacteria;Other;Other;Other;Other;Other* | 3.909 | 7.453 | 6.668 | 3.744 | 4.765 | 4.423 | 1.669 |
| *Alphaproteobacteria;Other;Other;Other* | 0.002 | 0.011 | 0.000 | 0.009 | 0.008 | 0.005 | 0.005 |
| *Devosia* | 0.003 | 0.006 | 0.000 | 0.002 | 0.008 | 0.003 | 0.003 |
| *Rhizobiales;Other;Other* | 0.002 | 0.004 | 0.020 | 0.003 | 0.008 | 0.006 | 0.007 |
| *Rhodobacteraceae;Other* | 0.000 | 0.005 | 0.040 | 0.000 | 0.000 | 0.007 | 0.017 |
| *Alcaligenes* | 0.051 | 0.054 | 0.020 | 0.056 | 0.048 | 0.038 | 0.015 |
| *Alcaligenaceae;Other* | 0.000 | 0.006 | 0.000 | 0.000 | 0.000 | 0.001 | 0.003 |
| *Alysiella* | 0.005 | 0.000 | 0.000 | 0.003 | 0.000 | 0.001 | 0.002 |
| *Bdellovibrio* | 0.000 | 0.000 | 0.040 | 0.000 | 0.000 | 0.007 | 0.018 |
| *Vampirovibrio* | 0.024 | 0.016 | 0.060 | 0.026 | 0.008 | 0.022 | 0.020 |
| *Desulfovibrionaceae;Other* | 0.040 | 0.120 | 0.100 | 0.031 | 0.072 | 0.060 | 0.038 |
| *Deltaproteobacteria;Other;Other;Other* | 0.002 | 0.010 | 0.040 | 0.013 | 0.032 | 0.016 | 0.016 |
| *Ruminobacter* | 0.000 | 0.000 | 0.000 | 0.000 | 0.000 | 0.000 | 0.000 |
| *Succinimonas* | 0.002 | 0.002 | 0.000 | 0.005 | 0.000 | 0.001 | 0.002 |
| *Succinivibrio* | 0.003 | 0.030 | 0.060 | 0.004 | 0.000 | 0.016 | 0.026 |
| *Cardiobacteriaceae;Other* | 0.000 | 0.004 | 0.000 | 0.005 | 0.000 | 0.001 | 0.002 |
| *Escherichia/Shigella* | 0.005 | 0.012 | 0.020 | 0.006 | 0.000 | 0.007 | 0.008 |
| *Legionella* | 0.000 | 0.001 | 0.020 | 0.000 | 0.000 | 0.004 | 0.009 |
| *Proteobacteria;Other;Other;Other;Other* | 0.094 | 0.084 | 0.080 | 0.115 | 0.167 | 0.090 | 0.036 |
| *Treponema* | 0.002 | 0.024 | 0.020 | 0.006 | 0.000 | 0.009 | 0.011 |
| *Puniceicoccaceae;Other* | 0.014 | 0.004 | 0.000 | 0.004 | 0.008 | 0.005 | 0.005 |
| *Verrucomicrobia;Other;Other;Other;Other* | 0.058 | 0.094 | 0.080 | 0.025 | 0.032 | 0.048 | 0.030 |
| *Subdivision5_genera_incertae_sedis* | 0.272 | 0.933 | 0.916 | 0.352 | 0.869 | 0.557 | 0.327 |
| *Unclassified;Other;Other;Other;Other;Other* | 0.003 | 0.075 | 0.020 | 0.019 | 0.008 | 0.021 | 0.029 |

| Note cow 4 was unwell during the sampling period for the GS diet and therefore no rumen samples could be retrieved. Please also note that these values were calculated from actual values obtained and do not therefore take into account missing values which means that data in Table 5 which take into account missing values are slightly different. Please also note that only general above 0.001% of total reads are shown in this table. |
| --- |
