# Supplementary material for: Characterization of the rumen lipidome and microbiome of steers fed a diet supplemented with flax and echium oil
Source: Microb Biotechnol. 2014 Sep 16;8(2):331–41. doi: 10.1111/1751-7915.12164 (PMC4353346; doi:10.1111/1751-7915.12164)
Supplement: Supplementary file 5 [file mbt20008-0331-sd5.docx]

**Table 4 Supplementary** Comparison of the bacteria (Genus level) present within the rumen of steers fed grass silage and sugar beet and flax oil (GSF diet). Data shown are % occurrences within the total reads. Only sequences occurring above 0.001% of total read abundance are shown.

|  |  | Steer number Average | | | | | SED |
| --- | --- | --- | --- | --- | --- | --- | --- |
|  | 1 | 2 | 3 | 4 | 5 | 6 |  |

| *Corynebacterium* | 0.018 | 0.017 | 0.014 | 0.000 | 0.002 | 0.000 | 0.008 | 0.008 |
| --- | --- | --- | --- | --- | --- | --- | --- | --- |
| *Microbacterium* | 0.000 | 0.000 | 0.000 | 0.000 | 0.006 | 0.000 | 0.001 | 0.002 |
| *Microbacteriaceae;Other* | 0.009 | 0.008 | 0.054 | 0.000 | 0.028 | 0.037 | 0.023 | 0.019 |
| *Atopobium* | 0.018 | 0.000 | 0.000 | 0.011 | 0.002 | 0.000 | 0.005 | 0.007 |
| *Eggerthella* | 0.009 | 0.008 | 0.000 | 0.011 | 0.009 | 0.000 | 0.006 | 0.004 |
| *Olsenella* | 0.457 | 0.225 | 0.136 | 0.239 | 0.209 | 0.273 | 0.257 | 0.099 |
| *Coriobacteriaceae;Other* | 0.264 | 0.326 | 0.313 | 0.218 | 0.167 | 0.186 | 0.246 | 0.060 |
| *Slackia* | 0.053 | 0.017 | 0.014 | 0.000 | 0.016 | 0.012 | 0.019 | 0.016 |
| *Bacteroidales;Other;Other* | 0.014 | 0.017 | 0.014 | 0.014 | 0.006 | 0.015 | 0.013 | 0.021 |
| *Porphyromonadaceae;Other* | 0.000 | 0.008 | 0.014 | 0.011 | 0.005 | 0.037 | 0.012 | 0.012 |
| *Prevotellaceae;Other* | 0.035 | 0.042 | 0.027 | 0.033 | 0.014 | 0.025 | 0.029 | 0.009 |
| *Prevotella* | 0.053 | 0.067 | 0.027 | 0.011 | 0.014 | 0.025 | 0.033 | 0.020 |
| *Bacteroidetes;Other;Other;Other;Other* | 0.026 | 0.017 | 0.000 | 0.000 | 0.008 | 0.025 | 0.013 | 0.011 |
| *Parachlamydiaceae;Other* | 0.000 | 0.000 | 0.000 | 0.011 | 0.003 | 0.000 | 0.002 | 0.004 |
| *Parachlamydia* | 0.000 | 0.008 | 0.014 | 0.011 | 0.016 | 0.012 | 0.010 | 0.005 |
| *Anaerolineaceae;Other* | 0.018 | 0.025 | 0.014 | 0.011 | 0.000 | 0.000 | 0.011 | 0.009 |
| *Fibrobacter* | 0.290 | 0.451 | 0.014 | 0.218 | 0.077 | 0.261 | 0.218 | 0.143 |
| *Bacillales;Other;Other* | 0.009 | 0.000 | 0.000 | 0.011 | 0.005 | 0.000 | 0.004 | 0.004 |
| *Paenibacillaceae 1;Other* | 0.009 | 0.025 | 0.000 | 0.011 | 0.002 | 0.000 | 0.008 | 0.009 |
| *Paenibacillus* | 0.026 | 0.000 | 0.000 | 0.000 | 0.005 | 0.000 | 0.005 | 0.010 |
| *Leuconostoc* | 0.009 | 0.000 | 0.000 | 0.000 | 0.002 | 0.000 | 0.002 | 0.003 |
| *Weissella* | 0.000 | 0.000 | 0.000 | 0.000 | 0.008 | 0.000 | 0.001 | 0.003 |
| *Lactobacillales;Other;Other* | 0.000 | 0.008 | 0.000 | 0.000 | 0.000 | 0.000 | 0.001 | 0.003 |
| *Streptococcus* | 0.009 | 0.000 | 0.014 | 0.000 | 0.006 | 0.000 | 0.005 | 0.005 |
| *Anaerosporobacter* | 0.035 | 0.058 | 0.027 | 0.044 | 0.028 | 0.124 | 0.053 | 0.034 |
| *Mogibacterium* | 0.105 | 0.125 | 0.068 | 0.033 | 0.097 | 0.062 | 0.082 | 0.031 |
| *Eubacterium* | 0.185 | 0.250 | 0.449 | 0.185 | 0.292 | 0.112 | 0.246 | 0.107 |
| *Eubacteriaceae;Other* | 0.000 | 0.017 | 0.000 | 0.022 | 0.025 | 0.025 | 0.015 | 0.011 |
| *Pseudoramibacter* | 0.000 | 0.017 | 0.000 | 0.000 | 0.011 | 0.000 | 0.005 | 0.007 |
| *Acetitomaculum* | 0.009 | 0.000 | 0.000 | 0.000 | 0.000 | 0.000 | 0.001 | 0.003 |
| *Blautia* | 0.193 | 0.100 | 0.218 | 0.131 | 0.245 | 0.199 | 0.181 | 0.050 |
| *Butyrivibrio* | 13.550 | 11.010 | 9.262 | 16.001 | 11.423 | 16.750 | 12.999 | 2.702 |
| *Clostridium XlVa* | 0.000 | 0.000 | 0.000 | 0.000 | 0.005 | 0.012 | 0.003 | 0.005 |
| *Clostridium XlVb* | 0.026 | 0.017 | 0.027 | 0.022 | 0.013 | 0.050 | 0.026 | 0.012 |
| *Coprococcus* | 0.228 | 0.100 | 0.123 | 0.272 | 0.294 | 0.112 | 0.188 | 0.079 |
| *Howardella* | 0.053 | 0.050 | 0.027 | 0.022 | 0.039 | 0.037 | 0.038 | 0.011 |
| *Lachnobacterium* | 0.026 | 0.042 | 0.082 | 0.011 | 0.019 | 0.062 | 0.040 | 0.025 |
| *Lachnospiracea_incertae_sedis* | 1.195 | 1.027 | 1.117 | 0.642 | 0.839 | 0.845 | 0.944 | 0.188 |
| *Lactonifactor* | 0.009 | 0.008 | 0.000 | 0.044 | 0.005 | 0.000 | 0.011 | 0.015 |
| *Moryella* | 0.501 | 0.593 | 0.831 | 0.544 | 0.750 | 0.422 | 0.607 | 0.141 |
| *Oribacterium* | 0.228 | 0.167 | 0.082 | 0.120 | 0.224 | 0.174 | 0.166 | 0.052 |
| *Lachnospiraceae;Other* | 46.722 | 48.489 | 52.288 | 47.970 | 55.449 | 46.508 | 49.571 | 3.244 |
| *Pseudobutyrivibrio* | 3.322 | 3.414 | 3.855 | 4.125 | 4.304 | 4.796 | 3.969 | 0.510 |
| *Roseburia* | 0.062 | 0.017 | 0.027 | 0.022 | 0.055 | 0.050 | 0.039 | 0.017 |
| *Shuttleworthia* | 0.000 | 0.017 | 0.000 | 0.011 | 0.000 | 0.000 | 0.005 | 0.007 |
| *Syntrophococcus* | 0.009 | 0.008 | 0.000 | 0.011 | 0.008 | 0.012 | 0.008 | 0.004 |
| *Clostridiales;Other;Other* | 10.773 | 12.588 | 12.422 | 13.465 | 11.506 | 11.394 | 12.025 | 0.893 |
| *Acetivibrio* | 0.044 | 0.050 | 0.014 | 0.022 | 0.016 | 0.050 | 0.032 | 0.016 |
| *Clostridium IV* | 0.606 | 0.459 | 0.599 | 0.283 | 0.300 | 0.224 | 0.412 | 0.153 |
| *Oscillibacter* | 0.018 | 0.000 | 0.041 | 0.000 | 0.003 | 0.000 | 0.010 | 0.015 |
| *Ruminococcaceae;Other* | 3.225 | 2.629 | 2.356 | 1.829 | 1.648 | 2.348 | 2.339 | 0.518 |
| *Papillibacter* | 0.000 | 0.000 | 0.014 | 0.000 | 0.000 | 0.000 | 0.002 | 0.005 |
| *Ruminococcus* | 0.685 | 0.843 | 0.191 | 0.294 | 0.255 | 0.497 | 0.461 | 0.238 |
| *Saccharofermentans* | 1.503 | 1.311 | 0.994 | 0.653 | 0.558 | 0.907 | 0.988 | 0.335 |
| *Pelospora* | 0.009 | 0.000 | 0.000 | 0.000 | 0.003 | 0.000 | 0.002 | 0.003 |
| *Clostridia;Other;Other;Other* | 1.028 | 0.768 | 0.695 | 0.893 | 0.621 | 0.634 | 0.773 | 0.146 |
| *Bulleidia* | 0.018 | 0.050 | 0.000 | 0.011 | 0.013 | 0.025 | 0.019 | 0.016 |
| *Catenibacterium* | 0.000 | 0.008 | 0.000 | 0.000 | 0.000 | 0.000 | 0.001 | 0.003 |
| *Erysipelotrichaceae;Other* | 0.105 | 0.134 | 0.027 | 0.033 | 0.052 | 0.075 | 0.071 | 0.038 |
| *Succiniclasticum* | 0.466 | 0.250 | 0.232 | 0.054 | 0.122 | 0.211 | 0.223 | 0.128 |
| *Selenomonadales;Other;Other* | 0.009 | 0.008 | 0.000 | 0.011 | 0.000 | 0.000 | 0.005 | 0.005 |
| *Veillonellaceae;Other* | 0.149 | 0.058 | 0.068 | 0.065 | 0.025 | 0.112 | 0.080 | 0.040 |
| *Selenomonas* | 0.026 | 0.025 | 0.027 | 0.022 | 0.031 | 0.025 | 0.026 | 0.003 |
| *Firmicutes;Other;Other;Other;Other* | 8.673 | 9.149 | 8.976 | 6.999 | 7.450 | 7.679 | 8.154 | 0.815 |
| *Victivallis* | 0.009 | 0.017 | 0.014 | 0.033 | 0.009 | 0.025 | 0.018 | 0.009 |
| *Bacteria;Other;Other;Other;Other;Other* | 3.893 | 3.898 | 3.446 | 3.755 | 2.173 | 3.864 | 3.505 | 0.616 |
| *Alphaproteobacteria;Other;Other;Other* | 0.018 | 0.017 | 0.259 | 0.000 | 0.005 | 0.025 | 0.054 | 0.092 |
| *Rhizobiales;Other;Other* | 0.018 | 0.000 | 0.000 | 0.000 | 0.005 | 0.000 | 0.004 | 0.006 |
| *Kaistia* | 0.000 | 0.000 | 0.000 | 0.011 | 0.000 | 0.000 | 0.002 | 0.004 |
| *Rhodobacter* | 0.000 | 0.000 | 0.000 | 0.011 | 0.005 | 0.000 | 0.003 | 0.004 |
| *Alcaligenes* | 0.035 | 0.067 | 0.014 | 0.011 | 0.097 | 0.050 | 0.046 | 0.030 |
| *Alcaligenaceae;Other* | 0.000 | 0.000 | 0.000 | 0.000 | 0.000 | 0.025 | 0.004 | 0.009 |
| *Limnohabitans* | 0.000 | 0.017 | 0.000 | 0.000 | 0.000 | 0.000 | 0.003 | 0.006 |
| *Sutterellaceae;Other* | 0.009 | 0.000 | 0.000 | 0.000 | 0.003 | 0.000 | 0.002 | 0.003 |
| *Betaproteobacteria;Other;Other;Other* | 0.000 | 0.008 | 0.000 | 0.000 | 0.002 | 0.000 | 0.002 | 0.003 |
| *Vampirovibrio* | 0.018 | 0.067 | 0.014 | 0.011 | 0.006 | 0.037 | 0.025 | 0.021 |
| *Bilophila* | 0.000 | 0.000 | 0.000 | 0.000 | 0.000 | 0.012 | 0.002 | 0.005 |
| *Desulfovibrionaceae;Other* | 0.070 | 0.067 | 0.041 | 0.022 | 0.030 | 0.025 | 0.042 | 0.019 |
| *Deltaproteobacteria;Other;Other;Other* | 0.009 | 0.008 | 0.000 | 0.000 | 0.002 | 0.012 | 0.005 | 0.005 |
| *Ruminobacter* | 0.000 | 0.000 | 0.027 | 0.000 | 0.011 | 0.000 | 0.006 | 0.010 |
| *Succinimonas* | 0.018 | 0.008 | 0.000 | 0.000 | 0.002 | 0.000 | 0.005 | 0.007 |
| *Succinivibrio* | 0.008 | 0.007 | 0.004 | 0.000 | 0.000 | 0.000 | 0.003 | 0.008 |
| *Escherichia/Shigella* | 0.009 | 0.000 | 0.000 | 0.000 | 0.011 | 0.012 | 0.005 | 0.005 |
| *Acinetobacter* | 0.000 | 0.000 | 0.014 | 0.000 | 0.002 | 0.000 | 0.003 | 0.005 |
| *Xanthomonadaceae;Other* | 0.018 | 0.000 | 0.000 | 0.000 | 0.000 | 0.000 | 0.003 | 0.007 |
| *Proteobacteria;Other;Other;Other;Other* | 0.097 | 0.117 | 0.041 | 0.098 | 0.063 | 0.124 | 0.090 | 0.029 |
| *Treponema* | 0.026 | 0.017 | 0.000 | 0.000 | 0.003 | 0.000 | 0.008 | 0.010 |
| *Puniceicoccaceae;Other* | 0.000 | 0.000 | 0.027 | 0.033 | 0.003 | 0.012 | 0.013 | 0.013 |
| *Verrucomicrobia;Other;Other;Other;Other* | 0.053 | 0.050 | 0.054 | 0.054 | 0.027 | 0.075 | 0.052 | 0.014 |
| *Subdivision5_genera_incertae_sedis* | 0.518 | 0.442 | 0.218 | 0.250 | 0.169 | 0.249 | 0.308 | 0.127 |
| *Unclassified;Other;Other;Other;Other;Other* | 0.018 | 0.008 | 0.014 | 0.000 | 0.000 | 0.000 | 0.007 | 0.007 |
| *Corynebacterium* | 0.018 | 0.017 | 0.014 | 0.000 | 0.002 | 0.000 | 0.008 | 0.008 |
| *Microbacterium* | 0.000 | 0.000 | 0.000 | 0.000 | 0.006 | 0.000 | 0.001 | 0.002 |
| *Microbacteriaceae;Other* | 0.009 | 0.008 | 0.054 | 0.000 | 0.028 | 0.037 | 0.023 | 0.019 |
| *Atopobium* | 0.018 | 0.000 | 0.000 | 0.011 | 0.002 | 0.000 | 0.005 | 0.007 |
| *Eggerthella* | 0.009 | 0.008 | 0.000 | 0.011 | 0.009 | 0.000 | 0.006 | 0.004 |

|  |
| --- |

Please also note that these values were calculated from actual values obtained and do not therefore take into account missing values which means that data in Table 5 which take into account missing values are slightly different. Please also note that only general above 0.001% of total reads are shown in this table.
