# Supplementary material for: Characterization of the rumen lipidome and microbiome of steers fed a diet supplemented with flax and echium oil
Source: Microb Biotechnol. 2014 Sep 16;8(2):331–41. doi: 10.1111/1751-7915.12164 (PMC4353346; doi:10.1111/1751-7915.12164)
Supplement: Supplementary file 6 [file mbt20008-0331-sd6.docx]

**Table 5 Supplementary** Comparison of the bacteria (Genus level) present within the rumen of steers fed grass silage/sugar beet and echium oil. Data shown are % occurrences within the total reads. Only sequences occurring above 0.001% of total read abundance are shown.

|  |  | Steer number Average | | | | | SED |
| --- | --- | --- | --- | --- | --- | --- | --- |
|  | 1 | 2 | 4 | 5 | 6 |  |  |

| *Corynebacterium* | 0.003 | 0.000 | 0.038 | 0.000 | 0.004 | 0.009 | 0.015 |
| --- | --- | --- | --- | --- | --- | --- | --- |
| *Microbacteriaceae;Other* | 0.006 | 0.012 | 0.005 | 0.019 | 0.009 | 0.010 | 0.005 |
| *Mycobacterium* | 0.011 | 0.018 | 0.006 | 0.000 | 0.018 | 0.011 | 0.007 |
| *Nocardiaceae;Other* | 0.003 | 0.000 | 0.002 | 0.000 | 0.000 | 0.001 | 0.001 |
| *Actinomycetales;Other;Other* | 0.000 | 0.018 | 0.009 | 0.000 | 0.007 | 0.007 | 0.007 |
| *Propionibacteriaceae;Other* | 0.003 | 0.006 | 0.000 | 0.000 | 0.000 | 0.002 | 0.002 |
| *Atopobium* | 0.000 | 0.024 | 0.021 | 0.019 | 0.011 | 0.015 | 0.009 |
| *Eggerthella* | 0.000 | 0.012 | 0.018 | 0.019 | 0.013 | 0.012 | 0.007 |
| *Olsenella* | 0.213 | 1.216 | 0.451 | 0.186 | 0.410 | 0.495 | 0.375 |
| *Coriobacteriaceae;Other* | 0.219 | 0.617 | 0.402 | 0.465 | 0.329 | 0.406 | 0.133 |
| *Slackia* | 0.017 | 0.049 | 0.047 | 0.037 | 0.040 | 0.038 | 0.011 |
| *Bacteroidales;Other;Other* | 0.026 | 0.171 | 0.047 | 0.037 | 0.051 | 0.066 | 0.053 |
| *Porphyromonadaceae;Other* | 0.000 | 0.012 | 0.009 | 0.000 | 0.011 | 0.006 | 0.005 |
| *Paludibacter* | 0.000 | 0.012 | 0.001 | 0.000 | 0.000 | 0.003 | 0.005 |
| *Hallella* | 0.003 | 0.000 | 0.000 | 0.000 | 0.000 | 0.001 | 0.001 |
| *Prevotellaceae;Other* | 0.017 | 0.104 | 0.049 | 0.000 | 0.049 | 0.044 | 0.035 |
| *Prevotella* | 0.009 | 0.128 | 0.028 | 0.037 | 0.061 | 0.053 | 0.041 |
| *Bacteroidetes;Other;Other;Other;Other* | 0.020 | 0.061 | 0.026 | 0.000 | 0.023 | 0.026 | 0.020 |
| *Chlamydiales;Other;Other* | 0.000 | 0.012 | 0.002 | 0.000 | 0.000 | 0.003 | 0.005 |
| *Parachlamydia* | 0.011 | 0.000 | 0.005 | 0.000 | 0.000 | 0.003 | 0.004 |
| *Anaerolineaceae;Other* | 0.014 | 0.018 | 0.007 | 0.000 | 0.016 | 0.011 | 0.007 |
| *Chloroflexi;Other;Other;Other;Other* | 0.000 | 0.012 | 0.000 | 0.000 | 0.000 | 0.002 | 0.005 |
| *Elusimicrobium* | 0.000 | 0.000 | 0.006 | 0.000 | 0.000 | 0.001 | 0.002 |
| *Fibrobacter* | 0.145 | 0.825 | 0.385 | 0.242 | 0.314 | 0.382 | 0.235 |
| *Bacillales;Other;Other* | 0.003 | 0.000 | 0.001 | 0.000 | 0.007 | 0.002 | 0.003 |
| *Cohnella* | 0.006 | 0.000 | 0.000 | 0.037 | 0.000 | 0.009 | 0.014 |
| *Paenibacillaceae 1;Other* | 0.003 | 0.006 | 0.000 | 0.000 | 0.000 | 0.002 | 0.002 |
| *Paenibacillus* | 0.000 | 0.006 | 0.001 | 0.019 | 0.005 | 0.006 | 0.007 |
| *Thermoactinomyces* | 0.009 | 0.000 | 0.000 | 0.000 | 0.000 | 0.002 | 0.003 |
| *Lactobacillaceae;Other* | 0.000 | 0.006 | 0.002 | 0.000 | 0.002 | 0.002 | 0.002 |
| *Pediococcus* | 0.000 | 0.006 | 0.000 | 0.000 | 0.000 | 0.001 | 0.002 |
| *Leuconostoc* | 0.000 | 0.000 | 0.006 | 0.000 | 0.005 | 0.002 | 0.003 |
| *Weissella* | 0.003 | 0.024 | 0.009 | 0.093 | 0.007 | 0.027 | 0.034 |
| *Lactobacillales;Other;Other* | 0.000 | 0.000 | 0.001 | 0.000 | 0.005 | 0.001 | 0.002 |
| *Streptococcus* | 0.003 | 0.024 | 0.005 | 0.000 | 0.042 | 0.015 | 0.016 |
| *Anaerosporobacter* | 0.028 | 0.012 | 0.020 | 0.037 | 0.016 | 0.023 | 0.009 |
| *Helcococcus* | 0.000 | 0.000 | 0.002 | 0.019 | 0.000 | 0.004 | 0.007 |
| *Mogibacterium* | 0.125 | 0.079 | 0.075 | 0.000 | 0.069 | 0.070 | 0.040 |
| *Anaerofustis* | 0.000 | 0.006 | 0.005 | 0.037 | 0.005 | 0.011 | 0.013 |
| *Eubacterium* | 0.265 | 0.165 | 0.152 | 0.297 | 0.266 | 0.229 | 0.059 |
| *Eubacteriaceae;Other* | 0.017 | 0.018 | 0.022 | 0.000 | 0.027 | 0.017 | 0.009 |
| *Blautia* | 0.151 | 0.141 | 0.194 | 0.167 | 0.184 | 0.167 | 0.020 |
| *Butyrivibrio* | 8.136 | 7.662 | 10.853 | 9.147 | 8.968 | 8.953 | 1.094 |
| *Clostridium XlVa* | 0.006 | 0.000 | 0.000 | 0.000 | 0.002 | 0.001 | 0.002 |
| *Clostridium XlVb* | 0.048 | 0.037 | 0.013 | 0.000 | 0.004 | 0.020 | 0.019 |
| *Coprococcus* | 0.174 | 0.079 | 0.121 | 0.242 | 0.099 | 0.143 | 0.058 |
| *Howardella* | 0.026 | 0.000 | 0.022 | 0.000 | 0.022 | 0.014 | 0.011 |
| *Lachnobacterium* | 0.023 | 0.031 | 0.006 | 0.019 | 0.014 | 0.018 | 0.008 |
| *Lachnospiracea_incertae_sedis* | 0.652 | 1.069 | 0.545 | 0.688 | 0.900 | 0.771 | 0.189 |
| *Lactonifactor* | 0.009 | 0.000 | 0.005 | 0.000 | 0.005 | 0.004 | 0.003 |
| *Moryella* | 0.638 | 0.483 | 0.390 | 0.483 | 0.641 | 0.527 | 0.098 |
| *Oribacterium* | 0.048 | 0.073 | 0.109 | 0.074 | 0.126 | 0.086 | 0.028 |
| *Lachnospiraceae;Other* | 54.727 | 34.124 | 45.007 | 51.236 | 46.915 | 46.402 | 7.009 |
| *Pseudobutyrivibrio* | 2.311 | 1.827 | 2.791 | 3.328 | 2.610 | 2.574 | 0.499 |
| *Robinsoniella* | 0.003 | 0.000 | 0.001 | 0.000 | 0.005 | 0.002 | 0.002 |
| *Roseburia* | 0.040 | 0.000 | 0.015 | 0.037 | 0.036 | 0.026 | 0.016 |
| *Shuttleworthia* | 0.003 | 0.006 | 0.004 | 0.000 | 0.004 | 0.003 | 0.002 |
| *Syntrophococcus* | 0.006 | 0.000 | 0.007 | 0.000 | 0.005 | 0.004 | 0.003 |
| *Clostridiales;Other;Other* | 14.746 | 12.861 | 13.668 | 13.869 | 13.195 | 13.668 | 0.644 |
| *Acetivibrio* | 0.034 | 0.238 | 0.118 | 0.056 | 0.047 | 0.099 | 0.076 |
| *Clostridium IV* | 0.327 | 1.747 | 0.698 | 0.260 | 0.739 | 0.754 | 0.532 |
| *Flavonifractor* | 0.003 | 0.000 | 0.001 | 0.000 | 0.004 | 0.002 | 0.001 |
| *Oscillibacter* | 0.009 | 0.018 | 0.027 | 0.000 | 0.022 | 0.015 | 0.010 |
| *Ruminococcaceae;Other* | 1.748 | 7.234 | 3.348 | 2.119 | 3.989 | 3.688 | 1.950 |
| *Papillibacter* | 0.003 | 0.006 | 0.001 | 0.000 | 0.004 | 0.003 | 0.002 |
| *Pseudoflavonifractor* | 0.000 | 0.006 | 0.000 | 0.000 | 0.002 | 0.002 | 0.002 |
| *Ruminococcus* | 0.327 | 1.674 | 0.813 | 0.558 | 0.856 | 0.846 | 0.456 |
| *Saccharofermentans* | 0.752 | 4.155 | 2.471 | 1.264 | 2.208 | 2.170 | 1.172 |
| *Pelospora* | 0.000 | 0.006 | 0.001 | 0.000 | 0.002 | 0.002 | 0.002 |
| *Clostridia;Other;Other;Other* | 0.703 | 1.747 | 1.147 | 0.967 | 0.972 | 1.107 | 0.350 |
| *Bulleidia* | 0.011 | 0.281 | 0.123 | 0.019 | 0.047 | 0.096 | 0.101 |
| *Catenibacterium* | 0.040 | 0.287 | 0.012 | 0.000 | 0.061 | 0.080 | 0.106 |
| *Erysipelotrichaceae;Other* | 0.068 | 0.299 | 0.184 | 0.056 | 0.081 | 0.138 | 0.093 |
| *Acidaminococcaceae;Other* | 0.000 | 0.000 | 0.004 | 0.000 | 0.002 | 0.001 | 0.001 |
| *Succiniclasticum* | 0.142 | 0.813 | 0.365 | 0.335 | 0.401 | 0.411 | 0.220 |
| *Veillonellaceae;Other* | 0.026 | 0.165 | 0.067 | 0.074 | 0.061 | 0.079 | 0.046 |
| *Schwartzia* | 0.000 | 0.000 | 0.006 | 0.000 | 0.004 | 0.002 | 0.003 |
| *Selenomonas* | 0.023 | 0.098 | 0.025 | 0.019 | 0.022 | 0.037 | 0.030 |
| *Firmicutes;Other;Other;Other;Other* | 9.488 | 11.102 | 9.863 | 9.760 | 9.661 | 9.975 | 0.577 |
| *Victivallis* | 0.020 | 0.012 | 0.016 | 0.000 | 0.038 | 0.017 | 0.012 |
| *Bacteria;Other;Other;Other;Other;Other* | 2.784 | 6.599 | 4.258 | 2.919 | 4.368 | 4.186 | 1.373 |
| *Alphaproteobacteria;Other;Other;Other* | 0.003 | 0.012 | 0.013 | 0.000 | 0.004 | 0.006 | 0.005 |
| *Devosia* | 0.003 | 0.012 | 0.001 | 0.000 | 0.007 | 0.005 | 0.004 |
| *Alcaligenes* | 0.068 | 0.049 | 0.038 | 0.056 | 0.060 | 0.054 | 0.010 |
| *Burkholderiales;Other;Other* | 0.006 | 0.000 | 0.001 | 0.000 | 0.000 | 0.001 | 0.002 |
| *Vampirovibrio* | 0.026 | 0.000 | 0.004 | 0.093 | 0.029 | 0.030 | 0.033 |
| *Desulfovibrionaceae;Other* | 0.014 | 0.116 | 0.043 | 0.000 | 0.036 | 0.042 | 0.040 |
| *Deltaproteobacteria;Other;Other;Other* | 0.003 | 0.012 | 0.005 | 0.000 | 0.007 | 0.005 | 0.004 |
| *Succinivibrionaceae;Other* | 0.009 | 0.000 | 0.000 | 0.000 | 0.000 | 0.002 | 0.003 |
| *Succinimonas* | 0.000 | 0.000 | 0.010 | 0.000 | 0.004 | 0.003 | 0.004 |
| *Succinivibrio* | 0.003 | 0.012 | 0.015 | 0.000 | 0.002 | 0.006 | 0.006 |
| *Cardiobacteriaceae;Other* | 0.003 | 0.006 | 0.002 | 0.019 | 0.020 | 0.010 | 0.008 |
| *Escherichia/Shigella* | 0.003 | 0.000 | 0.007 | 0.000 | 0.007 | 0.003 | 0.003 |
| *Luteimonas* | 0.000 | 0.000 | 0.000 | 0.019 | 0.000 | 0.004 | 0.007 |
| *Proteobacteria;Other;Other;Other;Other* | 0.085 | 0.061 | 0.088 | 0.037 | 0.085 | 0.071 | 0.020 |
| *Treponema* | 0.000 | 0.018 | 0.005 | 0.000 | 0.004 | 0.005 | 0.007 |

|  |
| --- |

Note cow 3 data was removed due to the low number of pyrosequences obtained (<2,000). Please also note that these values were calculated from actual values obtained and do not therefore take into account missing values which means that data in Table 5 which take into account missing values are slightly different. Please also note that only general above 0.001% of total reads are shown in this table.
